# The Specificity of EGF-Stimulated IQGAP1 Scaffold Towards the PI3K-Akt Pathway is Defined by the IQ3 motif

**DOI:** 10.1038/s41598-019-45671-5

**Published:** 2019-06-24

**Authors:** Mo Chen, Suyong Choi, Oisun Jung, Tianmu Wen, Christina Baum, Narendra Thapa, Paul F. Lambert, Alan C. Rapraeger, Richard A. Anderson

**Affiliations:** University of Wisconsin-Madison, School of Medicine and Public Health, Madison, WI USA

**Keywords:** Phosphoinositol signalling, Target identification

## Abstract

Epidermal growth factor receptor (EGFR) and its downstream phosphoinositide 3-kinase (PI3K) pathway are commonly deregulated in cancer. Recently, we have shown that the IQ motif-containing GTPase-activating protein 1 (IQGAP1) provides a molecular platform to scaffold all the components of the PI3K-Akt pathway and results in the sequential generation of phosphatidylinositol-3,4,5-trisphosphate (PI3,4,5P_3_). In addition to the PI3K-Akt pathway, IQGAP1 also scaffolds the Ras-ERK pathway. To define the specificity of IQGAP1 for the control of PI3K signaling, we have focused on the IQ3 motif in IQGAP1 as PIPKIα and PI3K enzymes bind this region. An IQ3 deletion mutant loses interactions with the PI3K-Akt components but retains binding to ERK and EGFR. Consistently, blocking the IQ3 motif of IQGAP1 using an IQ3 motif-derived peptide mirrors the effect of IQ3 deletion mutant by reducing Akt activation but has no impact on ERK activation. Also, the peptide disrupts the binding of IQGAP1 with PI3K-Akt pathway components, while IQGAP1 interactions with ERK and EGFR are not affected. Functionally, deleting or blocking the IQ3 motif inhibits cell proliferation, invasion, and migration in a non-additive manner to a PIPKIα inhibitor, establishing the functional specificity of IQ3 motif towards the PI3K-Akt pathway. Taken together, the IQ3 motif is a specific target for suppressing activation of the PI3K-Akt but not the Ras-ERK pathway. Although EGFR stimulates the IQGAP1-PI3K and -ERK pathways, here we show that IQGAP1-PI3K controls migration, invasion, and proliferation independent of ERK. These data illustrate that the IQ3 region of IQGAP1 is a promising therapeutic target for PI3K-driven cancer.

## Introduction

EGFR and its downstream PI3K pathway are commonly deregulated in many cancers, including breast and head and neck cancer (HNC), making them promising therapeutic targets^1–3^. However, direct inhibition of these kinases has yielded mixed results^4–6^. We have recently discovered that the multi-domain scaffolding protein IQGAP1 provides a novel molecular platform for the assembly of PI4P-, PI4,5P_2_-, and PI3,4,5P_3_-generating enzymes (PI4KIIIα, PIPKIα, and PI3K, respectively) into functional proximity for concerted and efficient generation of the PI3,4,5P_3_ lipid messenger^7^. The generated PI3,4,5P_3_ recruits PDK1/Akt kinases to the IQGAP1 complex and activates them. This finding indicates that IQGAP1 is a novel therapeutic target for cancer therapies.

**Figure 1:**
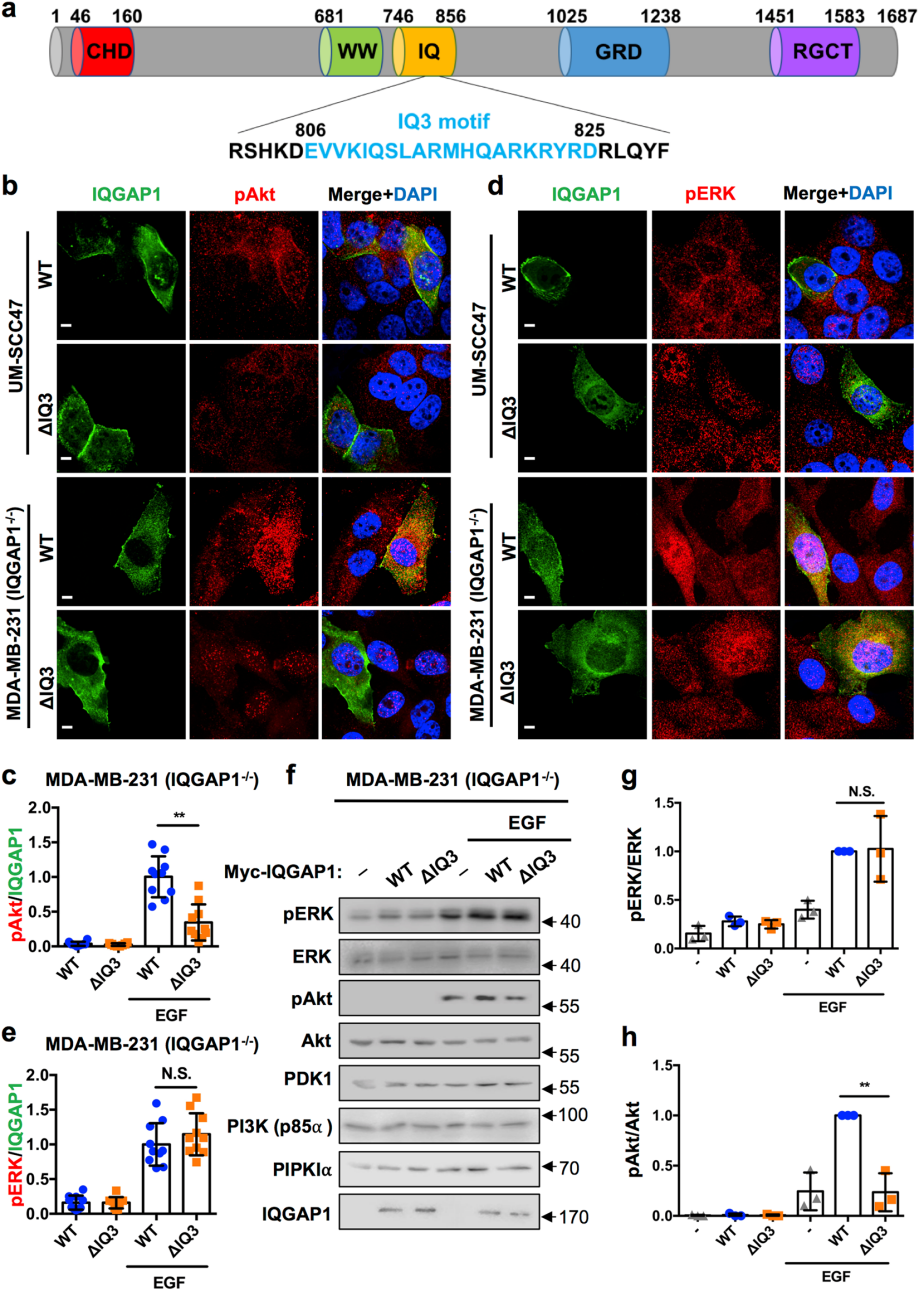
The IQ3 motif in IQGAP1 specifically mediates Akt but ERK activation. (**a**) Domain model of IQGAP1. (**b**–**e**) Deletion of the IQ3 motif in IQGAP1 blocked the inducible activation of the PI3K-Akt pathway but has no impact on the ERK pathway by immunofluorescent staining. UM-SCC47 and IQGAP1 null MDA-MB-231 cells post 24 h of transient transfection with GFP-tagged IQGAP1^WT/ΔIQ3^ constructs were starved for 24 h and then treated with 10 ng/ml EGF for 15 min. The cells were fixed and processed for immunofluorescent staining of pAkt (S473) or pERK. The nuclei were counterstained with DAPI. The images were taken by Leica SP8 confocal microscope and quantified by ImageJ. The signal intensity of pAkt/pERK (Red) channel divided by the signal intensity of IQGAP1 (Green) channel was used as the indicator for the impact of IQGAP1 WT and ΔIQ3 mutant on the PI3K-Akt/ERK pathway. **P < 0.01, n = 10. Error bars denote SD. Scale bar, 5 μm. The images of the starved cells were shown in Supplementary Figs 1 and 2. (**f**) IQ3 deletion in IQGAP1 lost the inducible function in the PI3K-Akt pathway but not the ERK pathway by WB. MDA-MB-231 (IQGAP1^−/−^) cells post 24 h of transient transfection with Myc-tagged IQGAP1^WT/ΔIQ3^ constructs were starved for 24 h and then treated with 10 ng/ml EGF for 15 min as indicated. The whole cell lysates were collected and processed for WB. Unprocessed images of the blots are shown in Supplementary Fig. 9. (**g**,**h**) Quantification of relative pERK and pAkt level in **f**. **P < 0.01, n = 3. Error bars denote SD.

IQGAP1 belongs to the IQGAP family together with IQGAP2 and IQGAP3^8^. IQGAP1 has five domains that are responsible for protein-protein interactions; the calponin homology domain (CHD), the WW domain, the IQ domain (IQ) consisting of four tandem IQ motifs, the RasGAP-related domain (GRD), and the RasGAP C-terminal domain (RGCT) (Fig. 1a). IQGAP1 functions as a scaffold, assembling crucial components of both the PI3K-Akt and Ras-ERK pathways^9^. This includes all PI3K-Akt pathway components (PI4KIIIα, PIPKIα, Ras, PI3K, PDK1, and Akt) as well as key Ras-ERK pathway components (Ras, Raf, MEK, and ERK)^10^. The Ras-ERK and PI3K-Akt pathways are key for controlling cell growth, survival, metabolism, and motility^11^. In response to agonist activation, these two pathways crosstalk to regulate downstream signaling pathways. Often, inhibition of one pathway results in the enhanced activation of the other and this has been argued to contribute to chemoresistance in cancer treatment^12^. For example, FOXO1, the downstream target of Akt, also binds to IQGAP1 and phosphorylated FOXO1 inhibits the Ras-ERK pathway^13^. The association between the PI3K-Akt and the Ras-ERK pathways coupled by IQGAP1 illustrates the importance of identifying specific sequences on IQGAP1 that selectively control the PI3K-Akt or Ras-ERK pathway.

The scaffolding of PI3K-Akt pathway components by IQGAP1 positions the phosphoinositide kinases into functional proximity for efficient generation of PI3,4,5P_3_ and Akt activation^7^. This suggests that each of the kinases binds to a dedicated sequence within IQGAP1. A key region is the IQ3 motif in the IQ domain (Fig. 1a). The IQ3 motif selectively binds to both PIPKIα and the p85α regulatory subunit of PI3K, which are essential components of the PI3K-Akt pathway^7^. However, whether the IQ3 sequence is specific for the PI3K pathway versus the ERK pathway has not been examined.

Here, we set out to determine if the IQ3 motif of IQGAP1 specifically regulates signaling through the PI3K-Akt pathway but not the Ras-ERK pathway. The results show that the IQ3 motif is required for PI3K-Akt but not Ras-ERK signaling. Deletion or blocking of the IQ3 motif resulted in the loss of interaction with the PI3K-Akt components while the binding to both ERK and cell surface EGFR is retained. Functionally, deletion or blocking of the IQ3 motif in IQGAP1 resulted in reduced cell proliferation, migration, and invasion in a similar but non-additive manner to PIPKIα inhibitor, suggesting a functional specificity of the IQ3 motif towards the PI3K-Akt pathway. Taken together, this work has demonstrated that the IQ3 motif is a specific target in IQGAP1 for the PI3K-Akt pathway.

## Results

### The IQ3 motif of IQGAP1 mediates the activation of Akt pathway

Ectopically expressed GFP- or Myc-tagged IQGAP1 fully rescues IQGAP1 deletion phenotypes^7,14,15^. To determine the role of the IQ3 motif in the activation of the PI3K-Akt pathway, human embryonic kidney (HEK293FT), human HNC (UM-SCC47, HPV-positive), and human breast carcinoma cells (MDA-MB-231) were transiently transfected with plasmids expressing GFP-tagged wild-type IQGAP1 (IQGAP1^WT^) or IQ3 (aa806–825)-deleted IQGAP1 (IQGAP1^∆IQ3^) (Fig. 1a) and Akt phosphorylation was measured in response to EGF stimulation. Compared to non-transfected cells, IQGAP1^WT^ enhanced Akt phosphorylation, whereas IQGAP1^∆IQ3^ failed to activate Akt (Fig. 1b and Supplementary Fig. 1). Quantification of the relative phospho-Akt intensity in the GFP-positive cells demonstrated a considerable reduction in Akt activation by IQGAP1^∆IQ3^ compared to IQGAP1^WT^ (Supplementary Fig. 1). These results were further confirmed using IQGAP1 knockout MDA-MB-231 cells and the HPV-negative UM-SCC1 HNC cells transfected with IQGAP1^WT^ or IQGAP1^∆IQ3^ expression plasmids (Fig. 1b,c and Supplementary Fig. 1**)**, indicating that the IQ3 sequence in IQGAP1 is required for Akt activation and this function is independent of HPV infection of the cells.

### Deletion of IQ3 motif in IQGAP1 does not affect Ras-ERK signaling

The IQ domain of IQGAP1 is shown to bind to the Ras-ERK components MEK1/2^16^ and ERK1/2^17^ and has been suggested to mediate the Ras-ERK signaling pathway^18^. To test this, ERK phosphorylation was analyzed by immunofluorescent staining in HEK293FT, UM-SCC1, UM-SCC47, and WT versus IQGAP1-null MDA-MB-231 cells ectopically expressing GFP-tagged IQGAP1^WT^ or IQGAP1^∆IQ3^. Deletion of IQ3 had no effect on ERK activation in all tested cells (Fig. 1d,e and Supplementary Fig. 2). In HEK293FT and UM-SCC1 cells, ERK phosphorylation was induced to a similar extent, irrespective of whether IQGAP1^WT^ or IQGAP1^∆IQ3^ was delivered to the cells (Supplementary Fig. 2), and the amount of ERK phosphorylation correlated with expression of each of these two IQGAP1 proteins (Supplementary Fig. 2). MDA-MB-231 cells exhibit higher endogenous levels of ERK activation due to their expression of a dominant active form of Ras^19^. Not surprisingly, transfection-mediated expression of either IQGAP1^WT^ or IQGAP1^∆IQ3^ in MDA-MB-231 cells did not significantly enhance ERK activation compared to untransfected cells (Supplementary Fig. 2). However, knocking out IQGAP1 in MDA-MB-231 cells re-sensitized them to ERK activation upon transfection-mediated expression of IQGAP1^WT^ or IQGAP1^∆IQ3^ (Fig. 1d,e), further demonstrating that the scaffolding role of IQGAP1 in the Ras-ERK pathway is independent of the IQ3 motif. Interestingly, in UM-SCC47 cells, transfection-mediated expression of either IQGAP1^WT^ or IQGAP1^∆IQ3^ failed to robustly enhance ERK activation compared to untransfected cells (Fig. 1d), suggesting a preferential scaffolding role of IQGAP1 for the PI3K-Akt pathway in this cell line. Together, the absence of a detectable difference in the effect of IQGAP1^WT^ versus IQGAP1^∆IQ3^ on ERK phosphorylation suggests that the IQ3 motif within IQGAP1 does not affect signaling through the ERK pathway.

ERK and Akt activation were further analyzed by immunoblotting. Consistent with immunofluorescence data in HEK293FT, UM-SCC1, and IQGAP1-null MDA-MB-231 cells, overexpression of IQGAP1^WT^ and IQGAP1^∆IQ3^ enhanced ERK phosphorylation by EGF treatment (Fig. 1f,g and Supplementary Fig. 3). Furthermore, transient ectopic expression of IQGAP1^WT^ or IQGAP1^∆IQ3^ in UM-SCC47 and MDA-MB-231 cells showed no difference in EGF-stimulated ERK activation (Supplementary Fig. 4). Importantly, Akt phosphorylation was significantly reduced in cells overexpressing IQGAP1^∆IQ3^ compared to IQGAP1^WT^ (Fig. 1f,h and Supplementary Figs 3 and 4). Taken together, our data demonstrate that in the tested cells the IQ3 motif mediates the PI3K-Akt but not the Ras-ERK pathway.

**Figure 2:**
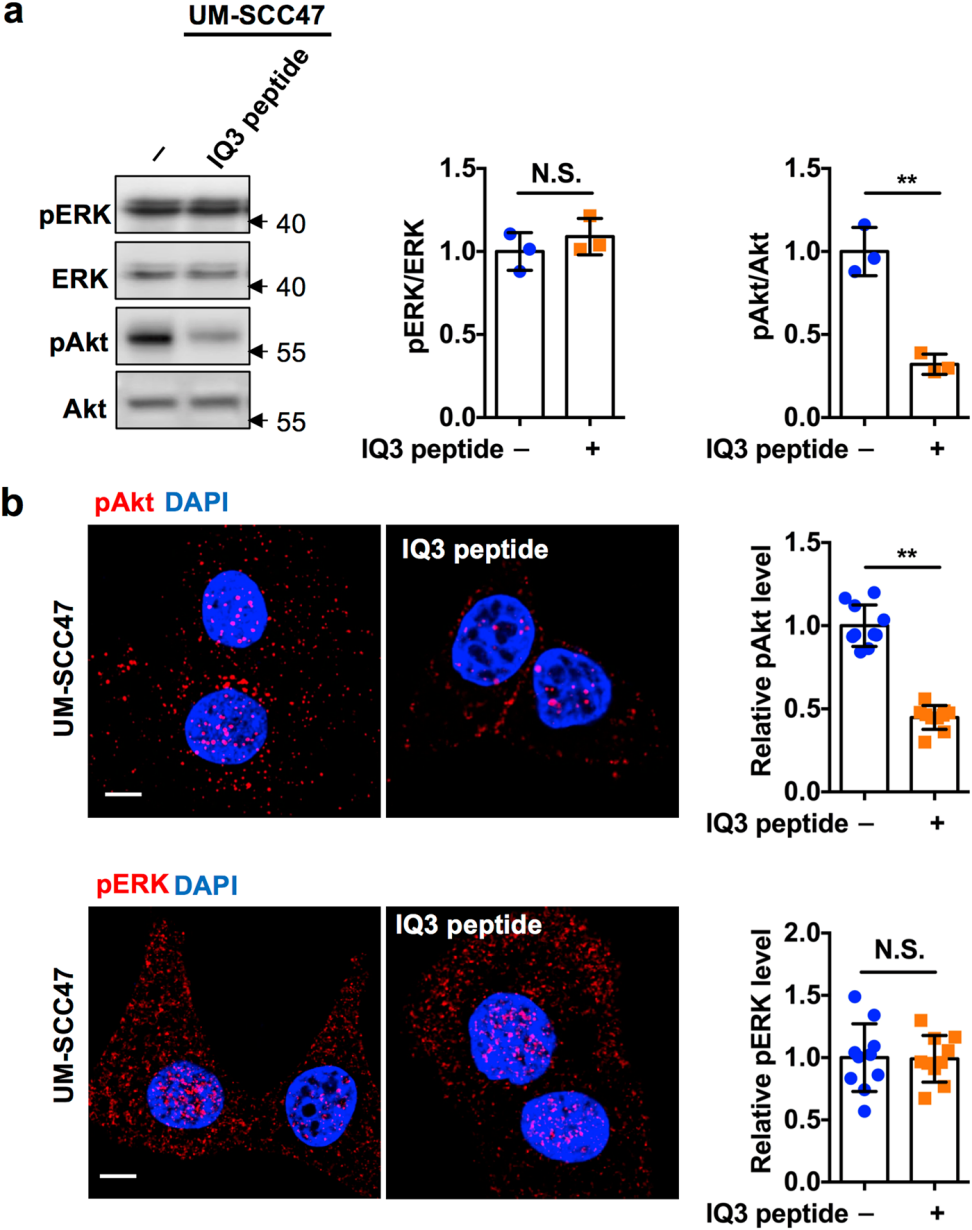
IQ3 peptide blocks Akt but not ERK activation in UM-SCC47 cells. (**a**) IQ3 peptide inhibited Akt but not ERK activation through WB. UM-SCC47 cells were treated with 30 μM IQ3 peptide for 48 h in DMEM with 10% FBS, and their whole cell lysates were analyzed by WB for Akt and ERK activation. **P < 0.01, n = 3. Error bars denote SD. Unprocessed images of the blots are shown in Supplementary Fig. 9. (**b**) IQ3 peptide inhibited Akt but not ERK activation through immunofluorescent staining. UM-SCC47 cells were treated with 30 μM IQ3 peptide for 48 h in DMEM with 10% FBS and fixed for immunofluorescent staining against pAkt (S473) or pERK. The nuclei were counterstained with DAPI. The images were taken by Leica SP8 confocal microscope and quantified by ImageJ. The signal intensity of pAkt/pERK (Red) channel was used as the indicator for the impact of IQ3 peptide on the PI3K-Akt/ERK pathway. **P < 0.01, n = 10. Error bars denote SD. Scale bar, 5 μm.

To explore how the IQ3 motif modulates the PI3K-Akt but not the Ras-ERK pathway, a short-length peptide derived from the IQ3 motif sequence of IQGAP1 (IQ3 peptide) was utilized^7^. Treatment with IQ3 peptide significantly reduced Akt but not ERK phosphorylation in UM-SCC47 cells analyzed either by immunoblotting (Fig. 2a) or *in situ* immunofluorescent staining (Fig. 2b), confirming that the IQ3 motif is responsible for Akt but not ERK activation.

### The IQ3 motif physically interacts with components of the PI3K-Akt but not with the Ras-ERK pathway

**Figure 3:**
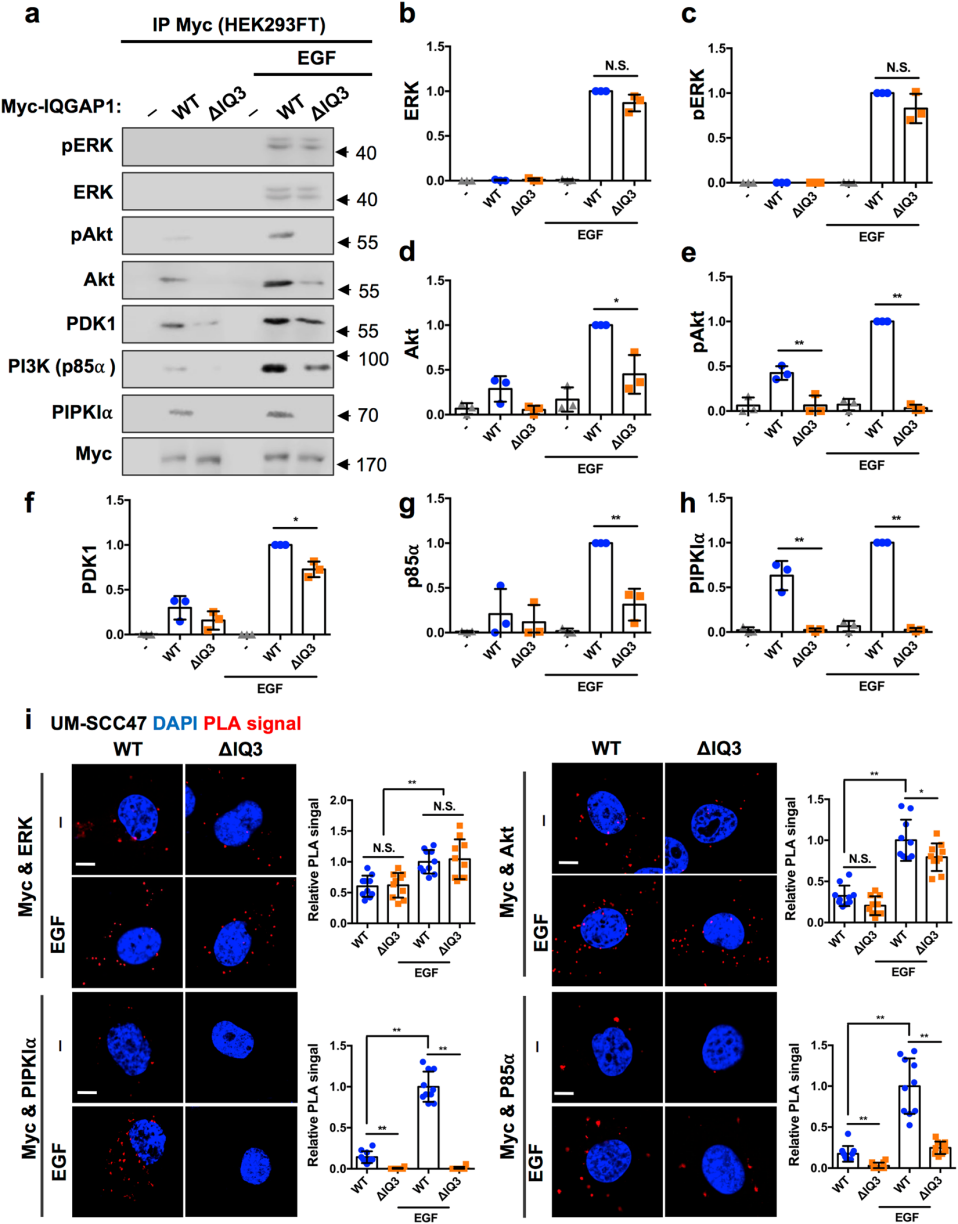
IQ3 motif specifically mediates the interactions between IQGAP1 and PI3K-Akt pathway components. (**a**) IQGAP1 WT and ΔIQ3 mutant associated ERK/PI3K-Akt pathway components were analyzed by immunoprecipitation. HEK293FT cells post 24 h of transient transfection with Myc-tagged IQGAP1^WT/ΔIQ3^ constructs were starved for 24 h and then treated with 10 ng/ml EGF for 15 min as indicated. The whole cell lysates were collected and processed for immunoprecipitation using anti-Myc antibody conjugated Protein A/G agarose beads. Unprocessed images of the blots are shown in Supplementary Fig. 9. (**b**–**h**) Quantification of immunoblots in (**a**). The whole cell lysates probed for WB as the input were shown in Supplementary Fig. 3a. *P < 0.05, **P < 0.01, n = 3. Error bars denote SD. (**i**) Deletion of IQ3 motif sustained the interaction of IQGAP1 with ERK but reduced the interaction of IQGAP1 with PI3K-Akt pathway components through PLA. UM-SCC47 cells post 24 h of transient transfection with Myc-tagged IQGAP1^WT^ or Myc-tagged IQGAP1^ΔIQ3^ constructs were starved for 24 h and then treated with 10 ng/ml EGF for 15 min. The cells were fixed and processed for PLA to determine the direct interaction between the Myc-tagged IQGAP1 WT/ΔIQ3 mutant and ERK/Akt/PIPKIα/p85α. **P < 0.01, n = 10. Error bars denote SD. Scale bar, 5 μm.

To study whether the IQ3 motif selectively associates with members of the PI3K-Akt pathway, co-immunoprecipitation studies were performed with IQGAP1^WT^ or IQGAP1^∆IQ3^. EGF stimulation enhanced the interaction between IQGAP1^WT^ and ERK and phospho-ERK, as well as PIPKIα, p85α, PDK1, Akt, and phospho-Akt (Fig. 3a–h), recapitulating prior reports that IQGAP1 mediates both the Ras-ERK and PI3K-Akt pathways downstream of receptor tyrosine kinase activation^7,18^. The binding of ERK and phospho-ERK was retained upon deletion of the IQ3 motif. However, there was a significant reduction in the binding of IQGAP1^∆IQ3^ to Akt, phospho-Akt, PDK1, p85α and PIPKIα compared to IQGAP1^WT^. The binding between PIPKIα and IQGAP1 was completely lost when the IQ3 motif was deleted while the binding between p85α and IQGAP1 was partially reduced. Consistently, there was also a complete loss of phospho-Akt. These results are consistent with previous studies showing that the IQ3 motif is the binding site for both PIPKIα and p85α^7^. However, p85α also binds to the WW domain consistent with its partial reduction in binding^7^.

Next, we investigated the agonist-stimulated interaction between IQGAP1 and components of the PI3K-Akt vs. ERK by proximity ligation assay (PLA). PLA is a powerful technique to measure a close interaction between two proteins *in situ*^20–22^. The resolution limit of conventional confocal microscopy is 200 nm^23^, while PLA detects two protein’s proximity within <40 nm which is generally accepted direct binding^24^. As a result, each PLA focus represents the site of direct interaction between the two target proteins. The PLA signals of Myc-tagged IQGAP1^WT^ with ERK, Akt, PIPKIα, and p85α were detectable under the starved condition and were further induced by EGF stimulation (Fig. 3i and Supplementary Fig. 5a). In contrast, whereas the PLA signal between IQGAP1^∆IQ3^ and ERK was retained, the PLA signal between IQGAP1^∆IQ3^ and PIPKIα was completely lost and the PLA signal between IQGAP1^∆IQ3^ and either p85α or Akt was partially lost. There was no detectable PLA signal in the mock-transfected cells, validating the specificity of the PLA signals (Supplementary Fig. 5b,c). Taken together, in multiple cells tested by a variety of methods, the IQ3 motif of IQGAP1 selectively interacts with PI3K-Akt pathway components but not with ERK, and this selective interaction fosters a specific activation of the PI3K-Akt pathway by the IQ3 motif.

### Deletion of the IQ3 motif retains the IQGAP1 binding to EGFR

**Figure 4:**
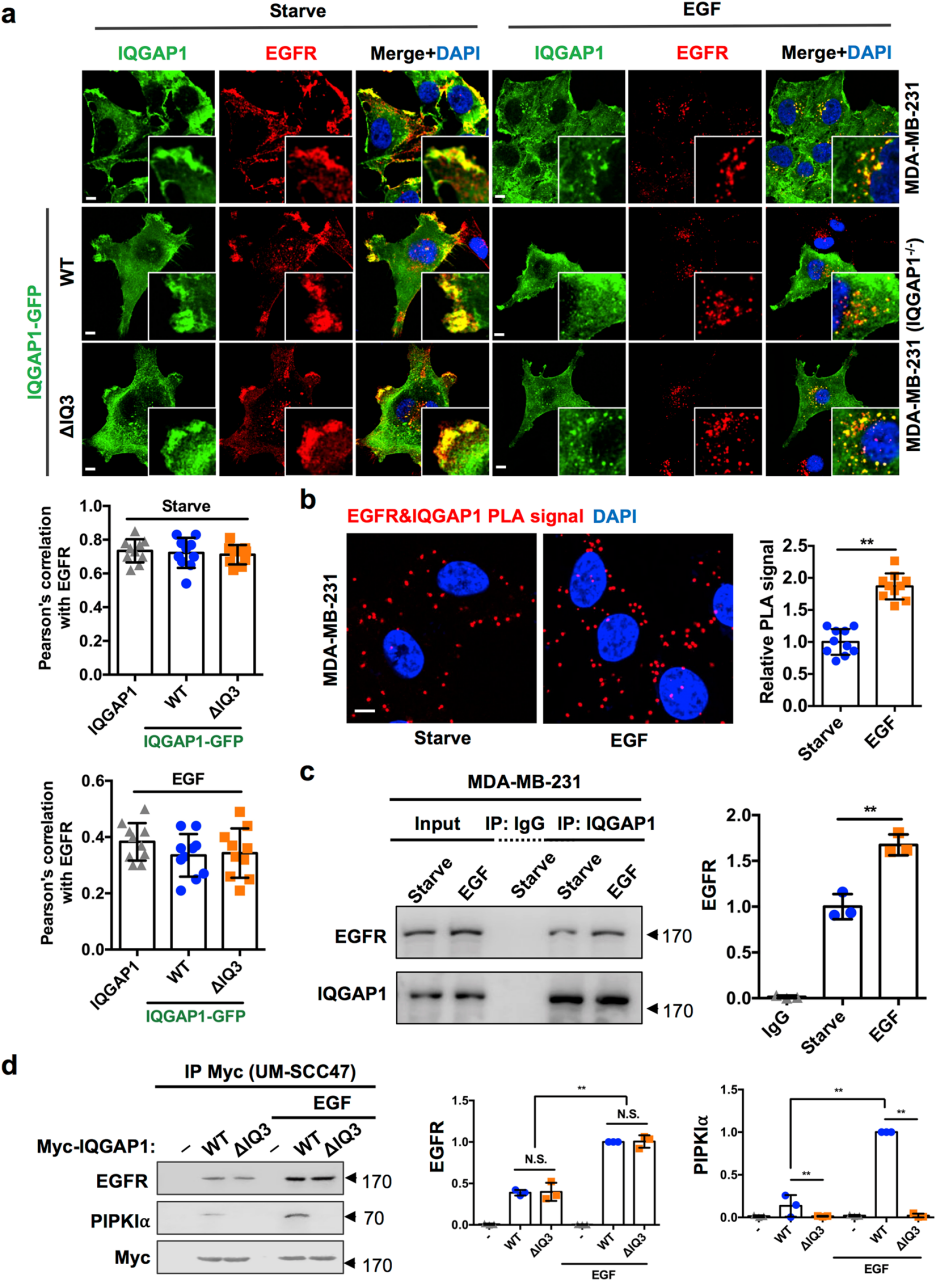
IQ3 deletion in IQGAP1 retains its association with EGFR. (**a**) IQGAP1 was colocalized with EGFR and the deletion of IQ3 motif in IQGAP1 sustained its colocalization with EGFR under starved and stimulated condition. Non-transfected MDA-MB-231 cells or IQGAP1-null MDA-MB-231 cells post 24 h of transient transfection with GFP-tagged IQGAP1^WT/ΔIQ3^ constructs were starved for 24 h and then treated with 10 ng/ml EGF for 15 min. The cells were fixed and processed for immunofluorescent staining of EGFR and IQGAP1. The nuclei were counterstained with DAPI. The images were taken by Leica SP8 confocal microscope. The colocalization of EGFR and endogenous/GFP-tagged IQGAP1 in the periphery of the starved cells or in the perinuclear region of the EGF-treated cells was quantified using Pearson’s correlation coefficient. n = 10. Error bars denote SD. Scale bar, 5 μm. (**b**) IQGAP1 interacted with EGFR and their interaction was induced by EGF treatment through PLA. MDA-MB-231 cells were starved for 24 h and then treated with 10 ng/ml EGF for 15 min. The cells were fixed and processed for PLA to determine the direct interaction between IQGAP1 and EGFR. **P < 0.01, n = 10. Error bars denote SD. Scale bar, 5 μm. (**c**) IQGAP1 was associated with EGFR and their association was induced by EGF treatment through immunoprecipitation. MDA-MB-231 cells were starved for 24 h and then treated with 10 ng/ml EGF for 15 min as indicated. The whole cell lysates were collected and processed for immunoprecipitation using anti-IQGAP1 antibody conjugated Protein A/G agarose beads. **P < 0.01, n = 3. Error bars denote SD. Unprocessed images of the blots are shown in Supplementary Fig. 9. (**d**) IQ3 deletion in IQGAP1 sustained the association with EGFR but not PIPKIα by immunoprecipitation. UM-SCC47 cells post 24 h of transient transfection with Myc-tagged IQGAP1^WT/ΔIQ3^ constructs were starved for 24 h and then treated with 10 ng/ml EGF for 15 min as indicated. The whole cell lysates were collected and processed for immunoprecipitation using anti-Myc antibody conjugated Protein A/G agarose beads. Unprocessed images of the blots are shown in Supplementary Fig. 9. **P < 0.01, n = 3. Error bars denote SD.

IQGAP1 functions to scaffold components of PI3K-Akt and Ras-ERK pathways upon agonist (e.g. EGF) stimulation^7,18^. IQGAP1 has been reported to bind to EGFR and modulate EGFR’s activation with the potential binding site being within the IQ domain of IQGAP1^25^. These reports suggest that the attenuation of PI3K-Akt pathway activation by IQ3 motif deletion may be due to the defective interaction with membrane receptors including EGFR. Therefore, it was important to examine whether the IQ3 motif mediates the binding of IQGAP1 to EGFR. For this, first, the colocalization between IQGAP1 and EGFR was quantified by co-immunofluorescent staining. Endogenous IQGAP1 in serum-starved MDA-MB-231 cells or transfected GFP-tagged IQGAP1^WT^ and IQGAP1^∆IQ3^ in serum-starved IQGAP1 null MDA-MB-231 cells were indistinguishably colocalized with endogenous EGFR at the periphery/surface of cells by quantification of the Pearson’s correlation coefficient (Fig. 4a). With EGF stimulation, endogenous IQGAP1 and transfected IQGAP1^WT^ and IQGAP1^∆IQ3^ were colocalized with EGFR at a similar level in the cytoplasm, presumably due to internalization. These results show that IQGAP1 is associated with EGFR and that deletion of the IQ3 motif in IQGAP1 did not mitigate its association with EGFR.

**Figure 5:**
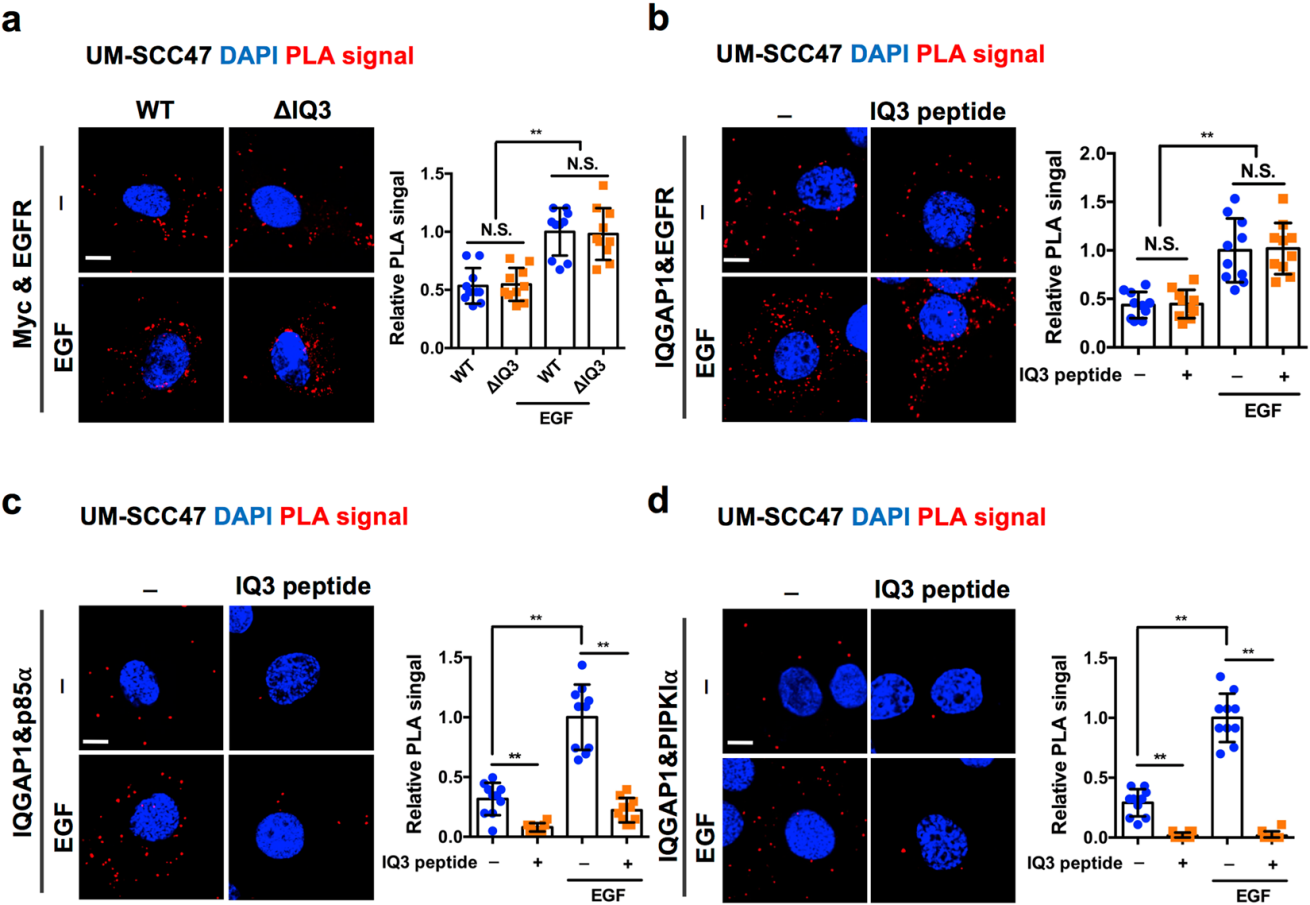
Deleting or blocking IQ3 motif in IQGAP1 sustains its interaction with EGFR in UM-SCC47 cells. (**a**) Deletion of IQ3 motif remained the interaction of IQGAP1 with EGFR through PLA. UM-SCC47 cells post 24 h of transient transfection with Myc-tagged IQGAP1^WT^ or Myc-tagged IQGAP1^ΔIQ3^ constructs were starved for 24 h and then treated with 10 ng/ml EGF for 15 min. The cells were fixed and processed for PLA to determine the direct interaction between the Myc-tagged IQGAP1 WT/ΔIQ3 mutant and EGFR. **P < 0.01, n = 10. Error bars denote SD. Scale bar, 5 μm. (**b**–**d**) IQ3 peptide did not affect the interaction between IQGAP1 and EGFR but blocked the interaction between IQGAP1 and p85α/PIPKIα. UM-SCC47 cells post 24 h starvation were treated with 30 μM IQ3 peptide for 3 h. After that, the cells were stimulated by 10 ng/ml EGF for 15 min before fixation. Then the cells were processed for proximity ligation assay (PLA) to detect the direct interaction between IQGAP1 and EGFR/p85α/PIPKIα. The nuclei were counterstained by DAPI. The images were taken by Leica SP8 confocal microscope and processed by ImageJ. **P < 0.01, n = 10. Error bars denote SD. Scale bar, 5 μm.

We next checked the interaction between the endogenous IQGAP1 and EGFR by PLA and co-immunoprecipitation. The results show that there is an association between endogenous IQGAP1 and EGFR in MDA-MB-231 cells in quiescent cells and the signal is induced by EGF treatment, indicating an interaction between endogenous IQGAP1 and EGFR (Fig. 4b,c). We further assayed the interaction between EGFR and either IQGAP1^WT^ or IQGAP1^∆IQ3^ by co-immunoprecipitation. IQGAP1^∆IQ3^ interacted with EGFR under starved and EGF-stimulated conditions in both UM-SCC47 and HEK293FT cells comparable to that of IQGAP1^WT^ (Fig. 4d and Supplementary Fig. 6a). In addition, similar PLA signals between EGFR and either IQGAP1^WT^ or IQGAP1^∆IQ3^ were observed in those cells, with no PLA signal detected in the mock-transfected cells (Fig. 5a and Supplementary Fig. 6b,c).

We further studied whether the IQ3 peptide would interfere with the binding of IQGAP1 with EGFR and PI3K-Akt pathway components. Consistently, IQGAP1 sustained its binding with EGFR when cells were treated with the IQ3 peptide but completely lost its binding to PIPKIα and partially lost its binding to p85α in UM-SCC47 cells (Fig. 5b–d). Together these results suggest that the IQ3 motif is not the binding site for EGFR on IQGAP1, and the reduction of PI3K-Akt signaling by IQ3 disruption is rather a consequence of defects in IQGAP1’s binding to components of the PI3K-Akt pathway downstream of EGFR.

### Selective inhibition of the PI3K-Akt pathway by disruption of the IQ3 motif is sufficient to suppress cancer cell proliferation, migration, and invasion

**Figure 6:**
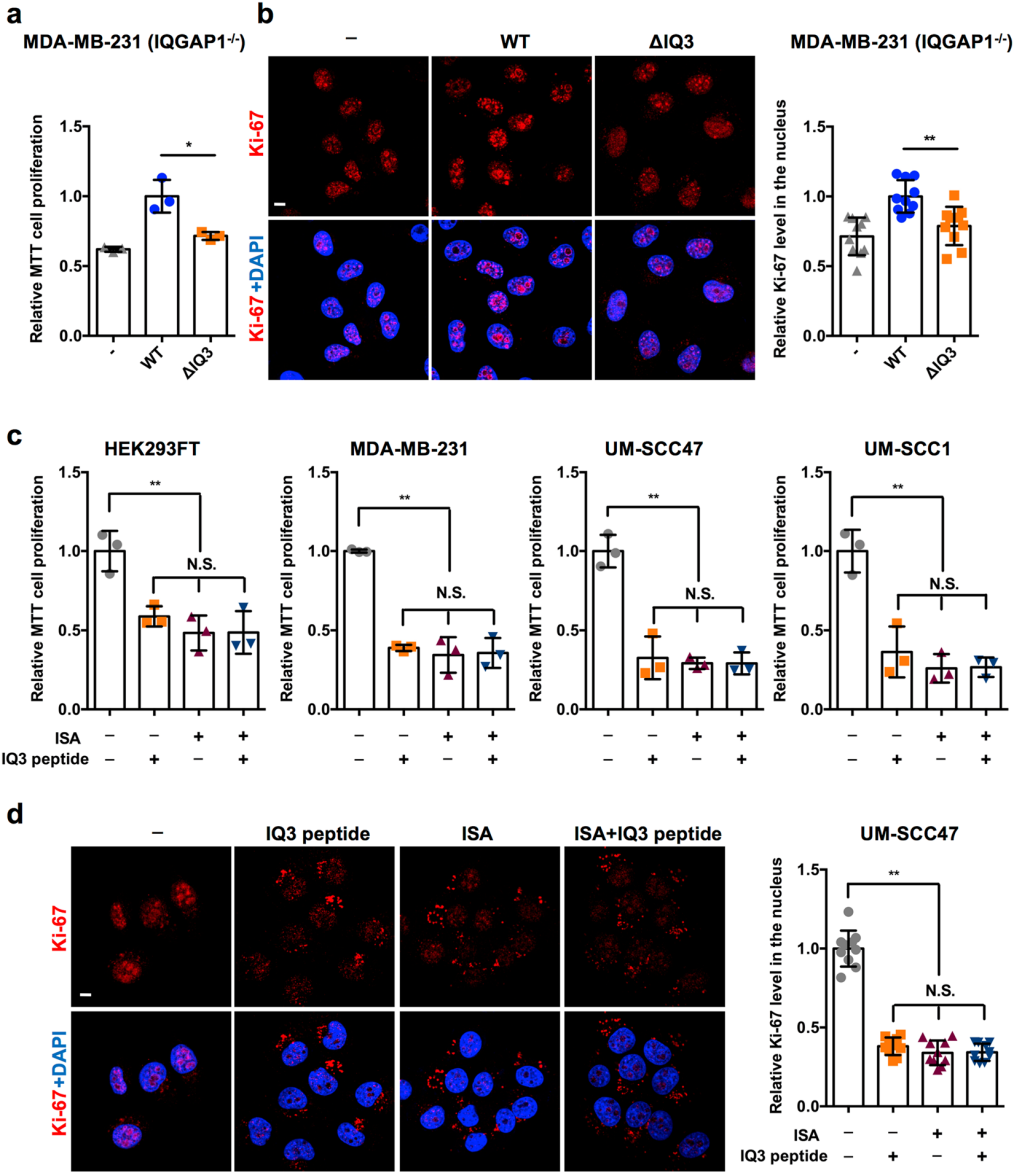
Deleting or blocking IQ3 motif in IQGAP1 reduces its inducible function in cell proliferation. (**a**) Deletion of IQ3 motif in IQGAP1 reduced its inducible function in cell proliferation through the MTT cell proliferation assay. MDA-MB-231 (IQGAP1^−/−^) cells post 48 h of transient transfection with Myc-tagged IQGAP1^WT/ΔIQ3^ constructs were processed for MTT cell proliferation assay. **P < 0.01, n = 3. Error bars denote SD. (**b**) Deletion of IQ3 motif in IQGAP1 reduced its inducible function on the level of nuclear Ki-67 (the marker of cell proliferation). MDA-MB-231 (IQGAP1^−/−^) cells post 48 h of transient transfection with Myc-tagged IQGAP1^WT/ΔIQ3^ constructs were processed for immunofluorescent staining against Ki-67. The nuclei were counterstained by DAPI. The images were taken by Leica SP8 confocal microscope. The nuclear Ki-67 level was quantified using ImageJ. **P < 0.01, n = 10. Error bars denote SD. Scale bar, 5 μm. (**c**) IQ3 peptide and PIPKIα inhibitor ISA inhibited cell proliferation in a non-additive manner through the MTT cell proliferation assay. HEK293FT, MDA-MB-231, UM-SCC47 or UM-SCC1 cells treated with 30 μM IQ3 peptide, 30 μM ISA, or the combination of them for 48 h were processed for MTT cell proliferation assay. **P < 0.01, n = 3. Error bars denote SD. (**d**) IQ3 peptide and PIPKIα inhibitor ISA inhibited cell proliferation in a non-additive manner through the immunofluorescent staining of Ki-67. UM-SCC47 cells treated with 30 μM IQ3 peptide, 30 μM ISA, or the combination of them for 48 h were processed for immunofluorescent staining against Ki-67. The nuclei were counterstained by DAPI. The images were taken by Leica SP8 confocal microscope. The nuclear Ki-67 level was quantified using ImageJ. **P < 0.01, n = 10. Error bars denote SD. Scale bar, 5 μm.

IQGAP1 functions as a scaffold protein in both the PI3K-Akt and Ras-ERK pathways that are the two most prominent signaling modules regulating cancer cell proliferation and migration^7,18,26,27^. Therefore, blockade of IQGAP1 is reported to suppress tumor progression in diverse cancer types^7,18,28–30^. We showed that disruption of IQ3 motif of IQGAP1 selectively inhibits the PI3K-Akt pathway (Figs 1, 2 and Supplementary Figs 1–4). Next, the role of IQ3 motif in IQGAP1-mediated cancer hallmarks was examined. We first tested the role of the IQ3 motif in cell proliferation. By the MTT proliferation assay, transient transfection of IQGAP1^WT^ promoted cell proliferation whereas IQGAP1^∆IQ3^ was reduced in its ability to promote the proliferation of HEK293FT, UM-SCC1, UM-SCC47, and WT/IQGAP1 null MDA-MB-231 cells, (Fig. 6a and Supplementary Fig. 7a). In addition, we monitored levels of Ki-67, a well-established proliferation marker^31^, by immunofluorescence. Transient overexpression of IQGAP1^WT^ significantly increased the nuclear level of Ki-67 compared to mock-transfected cells, whereas IQGAP1^∆IQ3^ failed to do so (Fig. 6b). These data indicate that the IQ3 motif is required for IQGAP1-mediated cancer cell proliferation.

To further examine the role of the IQ3 motif in cell proliferation, cells were treated with the IQ3 peptide or a PIPKIα inhibitor ISA-2011B (ISA)^32^ and cell proliferation was measured. In HEK293FT, MDA-MB-231, UM-SCC1 and UM-SCC47 cells, treatment of IQ3 peptide inhibited cell proliferation to a similar level as ISA, and combinatorial treatment of the two inhibitors did not further enhance the inhibitory effect (Fig. 6c), suggesting that the IQ3 peptide and ISA block the same signaling pathway. This result supports the previous observation that IQ3 motif mediates the IQGAP1-scaffolded PIPKIα-PI3K-Akt pathway^7^. Consistently, in MDA-MB-231 and UM-SCC47 cells, the IQ3 peptide and ISA reduced the nuclear level of Ki-67 in a non-additive manner (Fig. 6d and Supplementary Fig. 7b), indicating that the IQ3 motif of IQGAP1 mediates cell proliferation through the PIPKIα-PI3K-Akt pathway. Interestingly, the IQ3 peptide, ISA, and the combination increased cytosolic levels of Ki-67, suggesting that they might inhibit cell proliferation by blocking nuclear importation of Ki-67 (Supplementary Fig. 7c).

**Figure 7:**
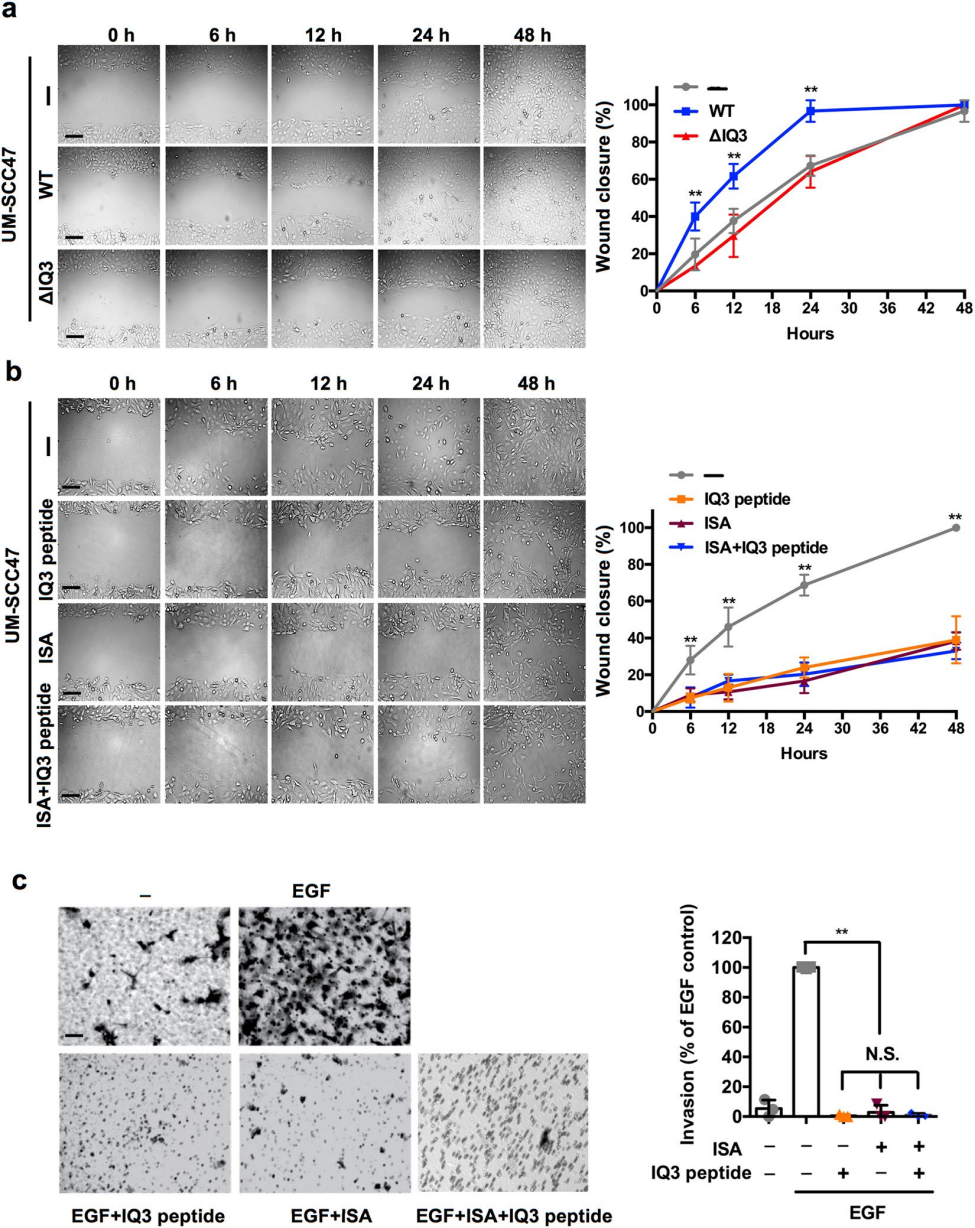
IQ3 motif in IQGAP1 mediates its inducible function in UM-SCC47 cell migration and invasion. (**a**) Deletion of IQ3 motif in IQGAP1 lost its inducible function in cell migration. The UM-SCC47 cells were seeded and transfected with Myc-tagged IQGAP1^WT/ΔIQ3^ constructs for 24 h and then grown to confluence. The cells were starved in serum-free medium for 24 h and then treated with 10 ng/ml EGF. The cellular layer in each plate was scratched using a plastic pipette tip. The migration of the cells at the edge of the scratch was imaged at 0, 6, 12, 24 and 48 h. Scale bar, 100 μm. **P < 0.01, n = 3. Error bars denote SD. (**b**) IQ3 peptide and PIPKIα inhibitor ISA inhibited cell migration in a non-additive manner. The confluent UM-SCC47 cells were starved in serum-free medium for 24 h and then treated with 30 μM IQ3 peptide, 30 μM ISA or the combination of them in the presence of 10 ng/ml EGF. The cellular layer in each plate was scratched using a plastic pipette tip. The migration of the cells at the edge of the scratch was imaged at 0, 6, 12, 24 and 48 h. Scale bar, 100 μm. **P < 0.01, n = 3. Error bars denote SD. (**c**) Blocking IQ3 motif inhibited cell invasion in a similar manner to PIPKIα inhibitor ISA. UM-SCC47 cell migration through LN332-coated filters in the presence of vehicle alone, vehicle plus 10 ng/ml EGF, or EGF containing 30 μM IQ3 peptide, 30 μM PIPKIα inhibitor ISA, or the combination of 30 μM IQ3 peptide and 30 μM PIPKIα inhibitor ISA. The empty pores with the size of 8 μm are showing up as white empty circles. The dark circles are pores containing cell processes. The migrated cells through the filters were black clusters on top of those pores with the size ranging from 20 to 100 μm. Only the migrated cells were quantified by ImageJ. Scale bar, 100 μm. **P < 0.01, n = 3. Error bars denote SD.

We next examined the role of the IQ3 motif in the inducible function of IQGAP1 during cell migration using the wound scratch assay. In UM-SCC47 and MDA-MB-231 cells, IQGAP1^WT^ enhanced EGF-stimulated wound closure compared to control, mock-transfected cells, whereas IQGAP1^∆IQ3^ had no impact (Fig. 7a and Supplementary Fig. 8a), indicating that the IQ3 motif is required for the IQGAP1-mediated cancer cell migration. In line with this, the IQ3 peptide inhibited the EGF-stimulated wound closure to a similar degree as the PIPKIα inhibitor, ISA, and the combinatorial treatment did not further enhance the inhibitory effect in UM-SCC47 and MDA-MB-231 cells (Fig. 7b and Supplementary Fig. 8b). These results demonstrated that the IQ3 motif is required for the inducible function of IQGAP1 in cell migration, and it mediates cell migration through the PI3K-Akt pathway.

EGFR coupled to the α3β1 and α6β4 integrins by syndecan-4 (Sdc4) plays a prominent role in the migration/invasion of epithelial cells on laminin-332 (LN332, comprised of α3β3γ2 chains, also known as laminin 5)^33–35^. Revisiting this role in the context of head and neck carcinoma cells, UM-SCC47 cells were observed to invade through LN332-coated filters in response to EGF with little to no invasion observed under serum-free conditions if EGF is not added (Fig. 7c). As IQGAP1 was shown to function downstream of agonist-stimulated EGFR and promote cell proliferation and migration, we asked whether the IQ3 motif in IQGAP1 mediates cell invasion. Blocking of the IQ3 motif in IQGAP1 by IQ3 peptide or inhibiting PIPKIα by ISA inhibited the EGF-stimulated invasion of UM-SCC47 cells to a similar degree (Fig. 7c). These results suggest that the IQ3 motif is specifically responsible for the scaffolding role of IQGAP1 towards the PI3K-Akt pathway that mediates the invasion of cancer cells.

## Discussion

**Figure 8:**
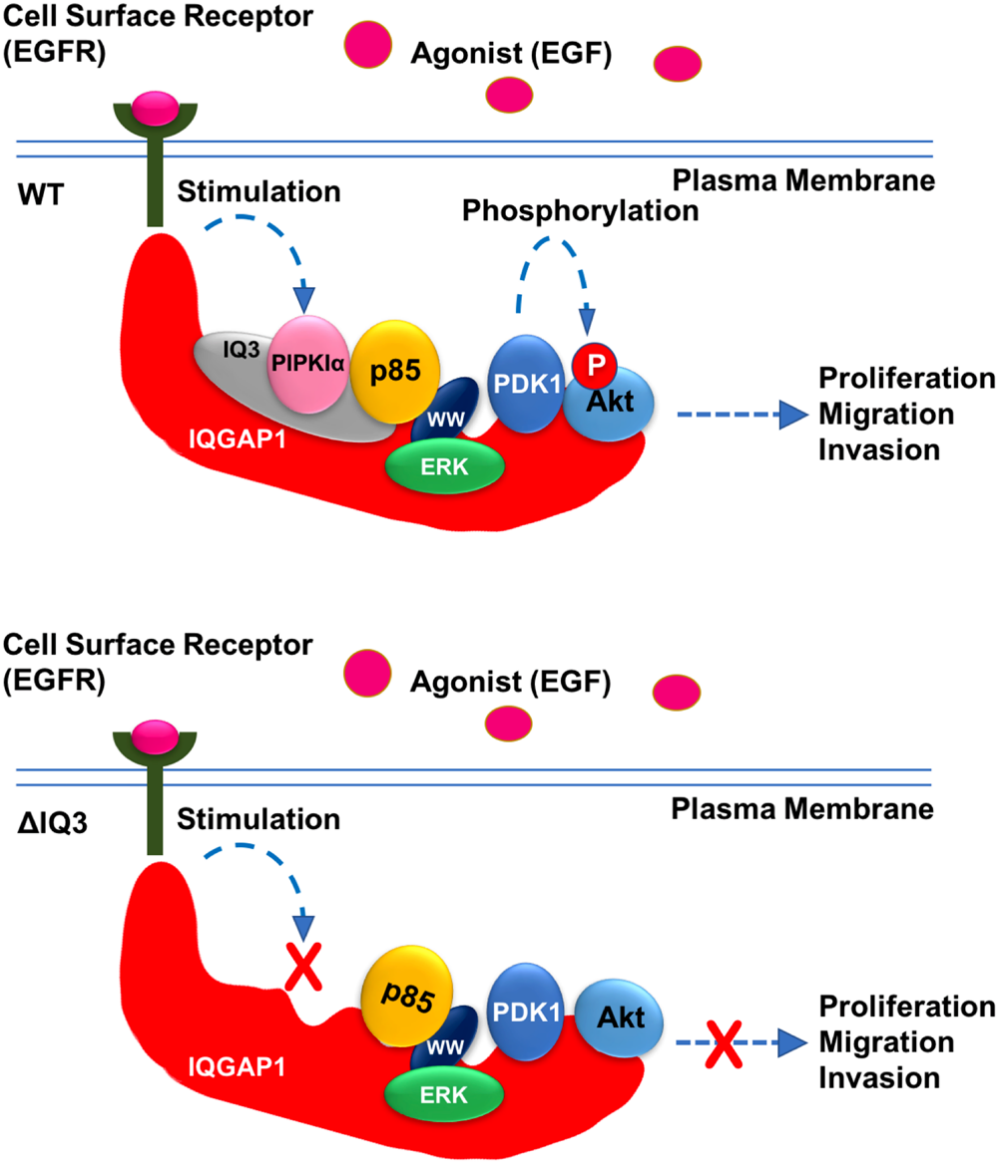
The proposed model for the scaffolding role of the IQ3 motif in IQGAP1 towards the PI3K-Akt pathway. The wild-type IQGAP1 provides a molecular platform for the assembly of the PI3K-Akt pathway components into proximity for concerted and efficient generation of the PI3,4,5P_3_ lipid messenger and activation of Akt upon agonist stimulation, promoting cellular processes related to cancer, such as proliferation, migration, and invasion. The IQ3 motif mediates the binding of PIPKIα and partial binding of PI3K (p85α) to IQGAP1. Deletion of the IQ3 motif in IQGAP1 sustains the interaction with EGFR and ERK but losses the PIPKIα binding and reduces PI3K (p85α) association, which turn off the pipeline for PI3,4,5P_3_ generation and Akt activation, leading to the blockade of cell proliferation, migration, and invasion.

Here we show that the IQ3 domain of IQGAP1 is a specific sequence required for the scaffolding of PI3K-Akt pathway components PIPKIα and p85α within the context of full-length IQGAP1 (Fig. 8). Deletion or blocking of the IQ3 motif in IQGAP1 specifically blocked the agonist-stimulated PI3K-Akt pathway without impacting on the IQGAP1-scaffolded Ras-ERK pathway. These findings demonstrate the functional specificity of IQGAP1 towards the PI3K-Akt pathway through the IQ3 motif and further define the critical role of the IQGAP1-mediated PI3K-Akt pathway in invasion, migration, and proliferation.

The ubiquitously expressed scaffold protein IQGAP1 participates in diverse biological functions through binding with numerous interacting proteins^36^. The IQ domain serves as the binding region on IQGAP1 for multiple interaction proteins. In the PI3K-Akt pathway, PIPKIα binds to the IQ3 motif in the IQ domain of IQGAP1 while PI3K (p85α) binds with both the IQ3 motif and WW domain^7^. In the Ras-ERK pathway, the IQ domain binds with B-Raf^10^ and MEK1/2^16^. ERK1/2 is conventionally believed to bind with the WW domain of IQGAP1. However, a recent study raised controversy and suggests that the IQ domain is the binding region for ERK1/2^17^. In addition, the IQ domain also interacts with cell surface receptors, such as EGFR^25^ and human epidermal growth factor receptor 2 (HER2)^37^. However, it remains unstudied which motif in the IQ domain is important for these interactions. Here we show that the IQ3 deletion mutant sustains the EGF-stimulated activation of ERK1/2, which is the downstream signaling of cell surface EGFR, Raf and MEK1/2, indicating that the interactions of IQGAP1 with EGFR, Raf, MEK1/2, and ERK1/2 are not dependent on the IQ3 motif in the IQ domain. Indeed, deleting or blocking IQ3 motif in IQGAP1 retains the binding with EGFR and ERK1/2. This indicates that the Ras-ERK pathway components and cell surface receptors require distinct interaction sites on IQGAP1 for their function.

IQGAP1 plays a crucial role in cell growth and survival^8^. Cancer cells with overexpressed IQGAP1 show enhanced Akt activation^7^ and diminished ERK activation^16,38^. Our previous data suggest that many breast cancer cells become addicted to the IQGAP1-PI3K pathway for survival^7^. However, some cancer cells do not fully rely on a single pathway^9,13^, consistent with our observations. For example, UM-SCC1 cells respond to EGF-stimulation largely by activation of both Akt and ERK, while MDA-MB-231 and UM-SCC47 cells mainly respond to EGF-stimulation by activation of Akt with a marginal increase in ERK phosphorylation. In addition, the scaffolding role of IQGAP1 may switch between PI3K-Akt and Ras-ERK pathways under different cellular conditions^9,12,13^, which may serve as an underlying mechanism for drug resistance. By determining the specific binding site and regulatory mechanism of each pathway scaffolded by IQGAP1, we may block the switch of the modulation role of IQGAP1 and thereby develop more efficient, novel cancer therapies.

The IQGAP1 IQ3 deletion mutant characterized in this study lacks 20 amino acids in the IQ3 motif that is a shared binding region for PIPKIα and p85α. It is unlikely that PIPKIα and p85α bind to the identical sequence. Future directions may include the definition of the exact binding sites of PIPKIα and p85α within the IQ3 domain. With the knowledge of binding sites of the individual kinases, we could further investigate the assembly of the PI3K-Akt pathway complex by IQGAP1 which could also help us understand how IQGAP2 and IQGAP3 function to scaffold signaling pathways.

IQGAP1 is known to play a pivotal role in the control of cell proliferation, migration, and invasion^39^. In this work, we showed that the IQ3 motif mediates the inducible function of IQGAP1 in these cellular processes. In addition, blocking the IQ3 motif in IQGAP1 by IQ3 peptide reduces cell proliferation, migration, and invasion in a similar but non-additive manner to that observed with a PIPKIα inhibitor, suggesting that the IQ3 motif mediates these cellular functions specifically through the PI3K-Akt pathway. As cell proliferation, migration, and invasion are fundamental processes in cancer progression and metastasis, knowledge of a specific region in IQGAP1 important in mediating PI3K-Akt pathway’s stimulation of those cellular processes provides a potential new target for developing novel cancer therapeutics.

Notably, the functional specificity of the IQ3 motif towards the PI3K-Akt pathway is induced upon agonist stimulation of cell surface EGFR. In addition to EGFR, IQGAP1 also binds with other cell surface receptors, including HER2^37^, C-X-C motif chemokine receptor 2 (CXCR2)^40^, fibroblast growth factor receptor 1 (FGFR1)^41^, nerve growth factor receptor (NGFR)^42^, and N-methyl-D-aspartate receptor (NMDAR)^43^. Therefore, future studies may test whether the IQ3 motif functions downstream of other cell surface receptors and broadly defines its functional specificity towards the PI3K-Akt or Ras-ERK pathway.

In summary, this work has demonstrated the IQ3 motif as a specific target in IQGAP1 for the PI3K-Akt pathway. This region is selectively responsible for the scaffolding role of IQGAP1 to assemble PIPKIα and PI3K and the full PI3K-Akt pathway, and this then functionally mediates cell proliferation, migration, and invasion specifically through the PI3K-Akt pathway. This discovery opens the door for a novel therapeutic strategy targeting the PI3K-Akt signaling in cancers.

## Materials and Methods

### Cell culture and reagents

All cells were purchased from ATCC and maintained in DMEM supplemented with 10% fetal bovine serum (FBS) (Thermo Fisher Scientific, Waltham, MA, USA). UM-SCC47 cells were further supplemented with 1 μg/ml hydrocortisone (Millipore Sigma, St. Louis, MO, USA). Antibodies were from Cell Signaling Technology (Danvers, MA, USA), including pAkt (Ser473, 4058), ERK (4695), pErk (Thr202/Tyr204, 4370), PDK1 (13037), PIPKIα (PIP5K1A, 9693), Santa Cruz Biotechnology (Dallas, TX, USA), including IQGAP1(SC-10792), EGFR (SC-120), Ki-67 (SC-23900), Abcam (Cambridge, MA, USA), including Akt (ab126811), p85α (PI3 kinase p85 alpha, ab191606), Millipore Sigma, including Myc-tag (05-724) and Myc-tag (C3956). Secondary antibodies were from Thermo Fisher Scientific or Santa Cruz Biotechnology. Lipofectamine^TM^ 3000 reagent for transfection and Oligo nucleotides were from Thermo Fisher Scientific. Other chemicals were from Millipore Sigma. The membrane penetrating the IQ3 peptide ((dR)(dR)(dR)(dR)(dR)(dR)(dR)(dR)EQKLISEEDLEVVKIQSLARMHQARKRYRD)^7^ as previously described were synthesized by Genscript. The PIPKIα inhibitor ISA-2011B was a gift from Jenny Persson (Lund University)^32^.

### Constructs

The GFP-tagged or Myc-tagged IQGAP1^WT^ construct was described previously^7,14^. The GFP-tagged or Myc-tagged IQGAP1^∆IQ3^ construct was generated by the overlapping polymerase chain reactions (PCR) to delete the 20 amino acids (EVVKIQSLARMHQARKRYRD) in the IQ3 motif within two XcmI sites. The forward linker 5′-CTGCGCTCCCACAAAGATCGCCTGCAGTACTTCCGG-3′ and reverse linker 5′-CCGGAAGTACTGCAGGCGATCTTTGTGGGAGCGCAG-3′ were used to link the flanking region of the deleted IQ3 motif. The upstream fragment of the IQ3 motif was amplified using the XcmI forward primer 5′-GAGTCCATGAACTTGGTGGACTTG-3′ and the reverse linker. The downstream fragment of the IQ3 motif was amplified using the forward linker and the XcmI reverse primer 5′-CCTCCCACCACAGAACTGGAGGA-3′. These fragments were joined by the linker region, amplified using XcmI forward/reverse primers, and cloned into the GFP-tagged or Myc-tagged IQGAP1^WT^ construct within the XcmI sites to replace the IQGAP1^WT^.

### Generation of IQGAP1 knockout MDA-MB-231 cell line

To stably knockout IQGAP1 expression in MDA-MB-231, CRISPR-Cas9 genome editing method was used^44^. Two guide RNA sequences, 5′-CCCGTCAACCTCGTCTGCGG-3′ and 5′-GGCGTGGCCCGGCCGCACTA-3′, that target the first exon of human IQGAP1 gene were cloned into PX459V2.0 vector (Addgene, Watertown, MA, USA). Constructs were transfected for 36 h and then transiently selected with 1 μg/ml puromycin (Millipore Sigma). After 48 h incubation, puromycin was removed and single cells were seeded in 96-well tissue culture dishes. Cells were expanded and positive colonies were selected by immunoblotting with specific antibodies against human IQGAP1. Successful editing of the IQGAP1 gene was further validated by genomic sequencing. Multiple clones of IQGAP1 knockout cells were tested to avoid clonal variation.

### Immunofluorescent staining

HEK293FT/MDA-MB-231 cells were seeded onto 0.2% Gelatin pre-coated glass coverslips of thickness No. 1.5 H. Cells post 24 h transient transfection of GFP-tagged IQGAP1^WT/∆IQ3^ were starved for 24 h in serum-free medium and stimulated with 10 ng/ml EGF for 15 min. Then the cells were fixed by 4% paraformaldehyde (PFA) in phosphate-buffered saline (PBS) and permeabilized with 0.3% Triton in PBS. the cells were then blocked with 3% Bovine Serum Albumin (BSA) in PBS for 1 h and incubated with primary antibody at 1:300 dilution in blocking buffer at 4 °C overnight. After washing 3 times by PBS, cells were stained with corresponding secondary antibody for 1 h. Cells were again washed 3 times before being mounted with Fluoroshield mounting buffer with DAPI (Vector Laboratories, Burlingame, CA, USA).

### Confocal microscopy

Images of fixed cells were collected by Leica SP8 confocal microscope (Leica Microsystems, Buffalo Grove, IL, USA), which includes a super-supercontinuum white-light laser for fluorescent excitation from 470 nm to 670 nm, a separate diode laser at 405 nm. This microscope is equipped with 3 Photomultiplier Tubes (PMTs) and 2 high-sensitive HyD detectors for image collection. All confocal images were acquired using the 100x objective lens (N.A. 1.4 oil). This microscope was taken and analyzed by Leica LAS software. The quantitative graph was generated by GraphPad Prism version 6.0 software (GraphPad Software, La Jolla, CA, USA).

### Immunoblotting and immunoprecipitation

HEK293FT cells post 24 h of transiently transfected with Myc-tagged IQGAP1^WT/∆IQ3^ constructs were starved for 24 h in serum-free medium and then stimulated with 10 ng/ml EGF for 15 min. Cells were lysed in a buffer containing 25 mM Tris-HCl pH 7.4, 150 mM NaCl, 1 mM EDTA, 1% NP-40, 5% glycerol, 5 mM Na_3_VO_4_, 20 mM NaF, and a protease inhibitor cocktail from Millipore Sigma. The protein concentration of lysates was measured by Bradford Protein Assay (Bio-Rad, Hercules, CA, USA), and an equal amount of protein was used for further analysis. All primary antibodies were at 1:1000 dilution for immunoblotting. For immunoprecipitation, 0.5 mg of proteins were incubated with 20 μl of anti-c-Myc antibodies pre-bound agarose for 2 h at 4 °C. After incubation, the agarose beads were washed 3 times with lysis buffer and eluted with SDS sample buffer. For WB, 10 μg of proteins were loaded for each lane. Protein bands were quantified by ImageJ, and the statistical analysis from three independent experiments was performed with GraphPad Prism version 6.0 software.

### Proximity ligation assay

UM-SCC47/HEK293FT cells were seeded to 0.2% Gelatin-coated glass cover slides, transiently transfected with the Myc-tagged IQGAP1^WT/∆IQ3^ constructs for 24 h and then starved for 24 h in serum-free medium and stimulated by 10 ng/ml EGF for 15 min. After that, the cells were fixed by 4% PFA and processed for PLA. The interaction between the Myc-tagged IQGAP1^WT/∆IQ3^ and EGFR/Akt/ERK/PIPKIα/p85α were determined by the PLA signals from Duolink *in situ* starter kit (Millipore Sigma). The images were collected by Leica SP8 confocal microscope and analyzed by ImageJ.

### MTT cell proliferation assay

In 96-well plates, 5 × 10^3^ cells/well were transfected with Myc-tagged IQGAP1^WT/∆IQ3^ constructs or treated with 30 μM IQ3 peptide/ISA for 48 h. After that, the cells were replaced with 100 μl of fresh medium plus 10 μl of the 12 mM MTT stock solution from the Vybrant^®^MTT cell proliferation assay kit (Thermo Fisher Scientific). After 4 hours incubation at 37 °C, all but 25 μl of medium was removed and the remains in the wells were mixed with 50 μl of DMSO. The mixture was incubated at 37 °C for 10 min, and the absorbance was read at 540 nm using the Synergy HTX Multi-Mode Microplate reader (BioTek Instruments Inc, Winooski, VT, USA).

### Wound healing assay

The UM-SCC47/MDA-MB-231 cells were transfected by IQGAP1^WT/∆IQ3^ constructs for 24 h before achieving confluence. For the IQ3 peptide/ISA treatment, UM-SCC47/MDA-MB-231 cells were cultured to reach confluency. Then the cells were starved in serum-free medium for 24 h and treated with 10 ng/ml EGF or in the combination of 30 μM IQ3 peptide/ISA as indicated. The cellular layer in each plate was scratched using a plastic pipette tip. The migration of the cells at the edge of the scratch was imaged at 0, 6, 12, 24 and 48 h using the Nikon Eclipse TE2000U microscope (Nikon Instruments Inc., Melville, NY, USA) and quantified by ImageJ.

### Invasion assay

The bottom polycarbonate filter surface of Transwell inserts (8 μm pores; Corning, Corning, NY, USA) was coated with 10 μg/ml of LN332 (Kerafast, Boston, MA, USA) diluted in PBS for 3 h at 37 °C. UM-SCC47 cells (5 × 10^4^) suspended in serum-free medium containing 1% BSA were plated in the upper insert chamber with or without 10 ng/ml EGF and indicated inhibitors. Cells were allowed to migrate/invade for 16 h at 37 °C. Cells on the bottom of the filter were then fixed with 4% PFA and stained with 0.1% Crystal Violet. Then the cells were imaged by the Nikon Eclipse TE2000U microscope and quantified by ImageJ.

### Statistical analysis

Data were expressed as the mean ± standard deviation ( ± SD). Statistical significance was determined using student t-test. *P < 0.05, **P < 0.01, n ≥ 3.

## Supplementary information

Supplementary information
