# Supplementary material for: The Specificity of EGF-Stimulated IQGAP1 Scaffold Towards the PI3K-Akt Pathway is Defined by the IQ3 motif
Source: Sci Rep. 2019 Jun 24;9:9126. doi: 10.1038/s41598-019-45671-5 (PMC6591252; doi:10.1038/s41598-019-45671-5)
Supplement: Supplementary file 1 — Supplementary information [file 41598_2019_45671_MOESM1_ESM.pdf]

Supplementary information of

The Specificity of EGF-Stimulated IQGAP1 Scaffold Towards the PI3K-Akt Pathway is Defined by the IQ3 motif

Mo Chen[1,2], Suyong Choi[1,2], Oisun Jung[1], Tianmu Wen[1], Christina Baum[1], Narendra Thapa[1], Paul F. Lambert[1], Alan C. Rapraeger[1], Richard A. Anderson[1,*]

1: University of Wisconsin-Madison, School of Medicine and Public Health, Madison, WI, USA.

2: Mo Chen and Suyong Choi are listed as the co-first authors

***Corresponding author:**
Richard A. Anderson, Ph.D.
3750 Medical Sciences Center
1300 University Ave
Madison, WI 53706
Ph. (608) 262-3753
E-mail: raanders@wisc.edu

Supplementary Fig. 1

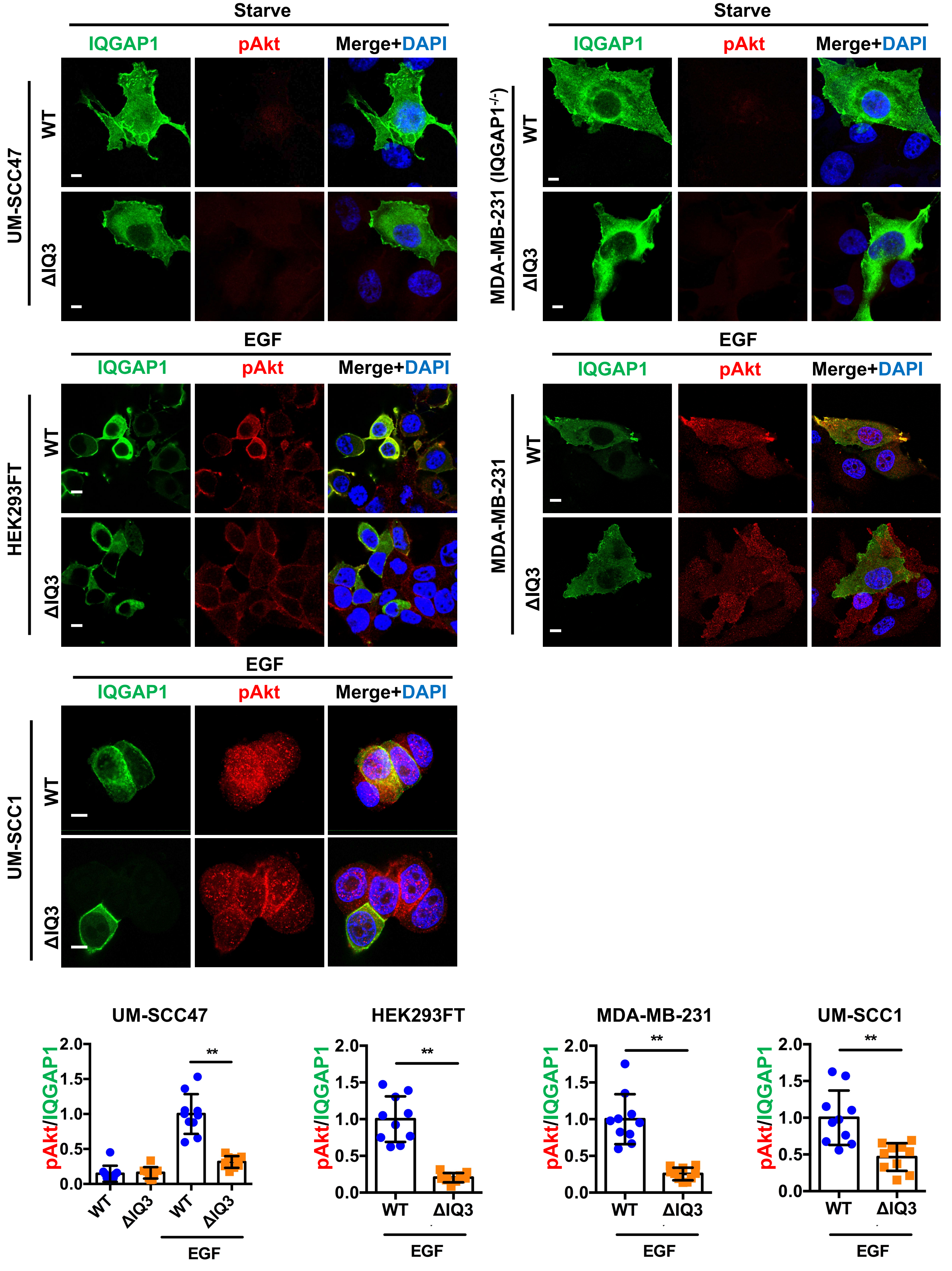

Supplementary Fig. 1 The IQ3 motif in IQGAP1 mediates the PI3K-Akt pathway.

Deletion of the IQ3 motif in IQGAP1 blocked the inducible activation of the PI3K-Akt pathway. UM-SCC47, IQGAP1 null MDA-MB-231, HEK293FT, MDA-MB-231, and UM-SCC1 cells post 24 h of transient transfection with GFP-tagged $IQGAP1^{WT}$ or GFP-tagged $IQGAP1^{\Delta IQ3}$ constructs were starved for 24 h and then treated with 10 ng/ml EGF for 15 min. The cells were fixed and processed for immunofluorescent staining of pAkt (S473). The nuclei were counterstained with DAPI. The images were taken by Leica SP8 confocal microscope and quantified by ImageJ. The signal intensity of pAkt (Red) channel divided by the signal intensity of IQGAP1 (Green) channel was used as the indicator for the effect of IQGAP1 WT and ΔIQ3 mutant on the PI3K-Akt pathway. ***P*<0.01, n=10. Error bars denote SD. Scale bar, 5 µm.

Supplementary Fig. 2

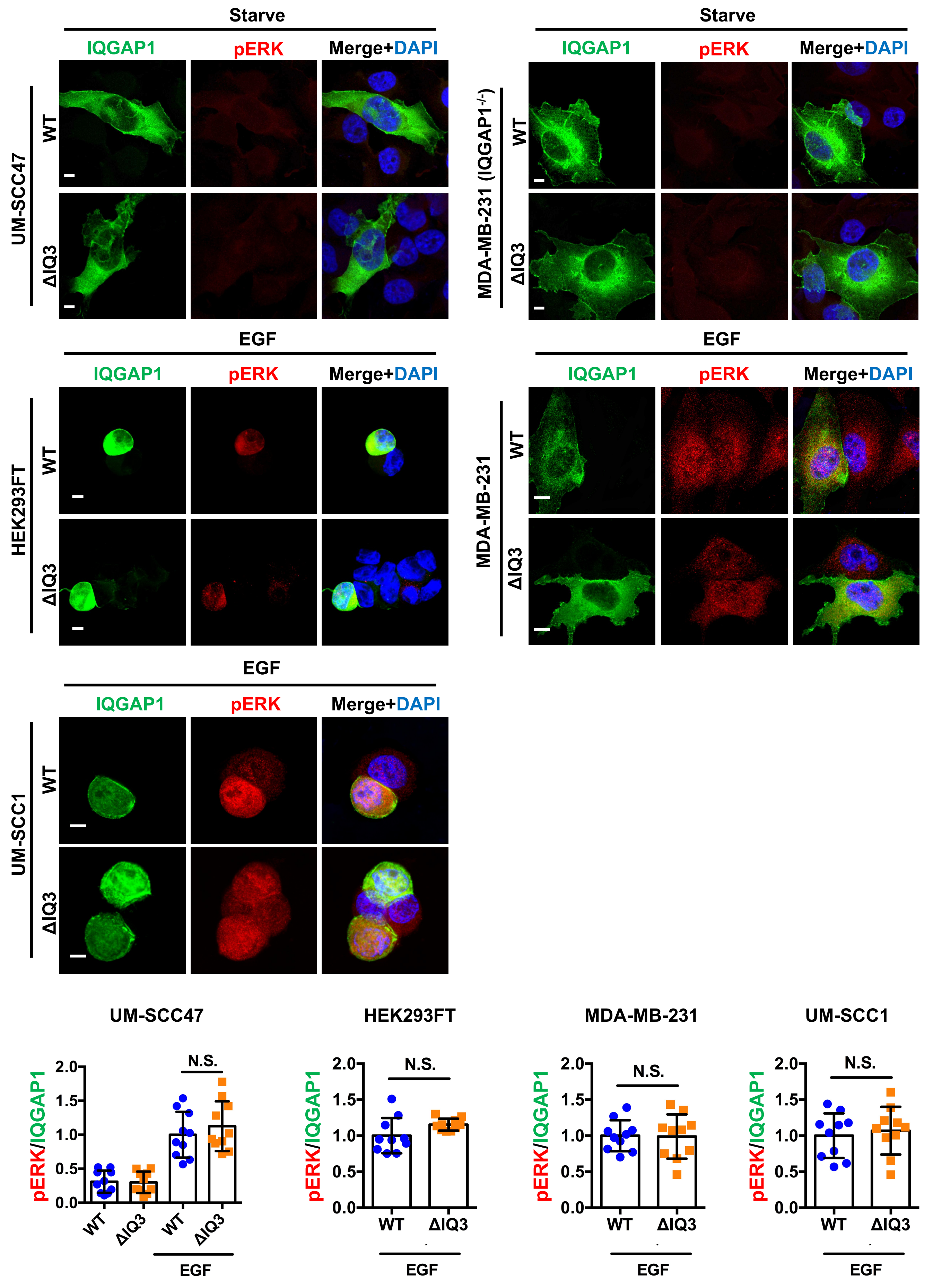

Supplementary Fig. 2 Deletion of the IQ3 motif in IQGAP1 has no impact on the ERK pathway.

Deletion of the IQ3 motif in IQGAP1 has no influence on the ERK activation. UM-SCC47, IQGAP1 null MDA-MB-231, HEK293FT, MDA-MB-231, and UM-SCC1 cells post 24 h of transient transfection with GFP-tagged IQGAP1^{WT} or GFP-tagged IQGAP1$^{\Delta IQ3}$ constructs were starved for 24 h and then treated with 10 ng/ml EGF for 15 min. The cells were fixed and processed for immunofluorescent staining of pERK. The nuclei were counterstained with DAPI. The images were taken by Leica SP8 confocal microscope and quantified by ImageJ. The signal intensity of pERK (Red) channel divided by the signal intensity of IQGAP1 (Green) channel was used as the indicator for the effect of IQGAP1 WT and ΔIQ3 mutant on the ERK pathway. n=10. Error bars denote SD. Scale bar, 5 µm.

Supplementary Fig. 3

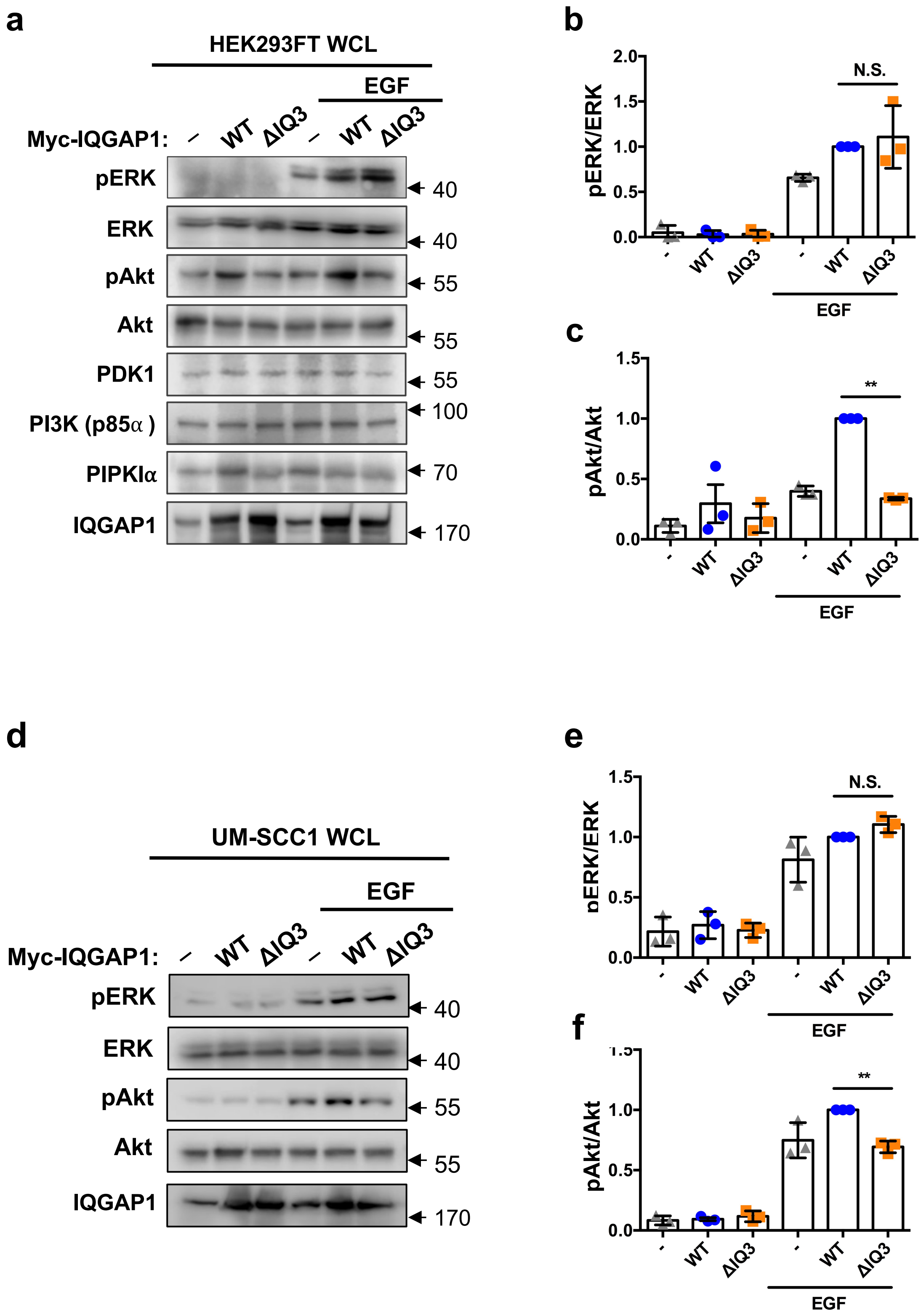

Supplementary Fig. 3 The IQ3 motif in IQGAP1 specifically mediates the PI3K-Akt pathway in HEK293FT and UM-SCC1 cells.

(**a**) EGF stimulated pAkt (S473) and pERK were measured in HEK293FT cells. HEK293FT cells post 24 h of transient transfection with Myc-tagged IQGAP1$^{WT/\Delta IQ3}$ constructs were starved for 24 h and then treated with 10 ng/ml EGF for 15 min as indicated. The whole cell lysates were collected and processed for WB. Unprocessed images of the blots are shown in **Supplementary Fig. 9**. (**b,c**) Quantification of relative pERK and pAkt level in **a**. (**d**) EGF stimulated pAkt (S473) and pERK were measured in UM-SCC1 cells. UM-SCC1 cells post 24 h of transient transfection with Myc-tagged IQGAP1$^{WT/\Delta IQ3}$ constructs were starved for 24 h and then treated with 10 ng/ml EGF for 15 min as indicated. The whole cell lysates were collected and processed for WB. Unprocessed images of the blots are shown in **Supplementary Fig. 9**. (**e,f**) Quantification of relative pERK and pAkt level in **d**. ***P*<0.01, n=3. Error bars denote SD.

Supplementary Fig. 4

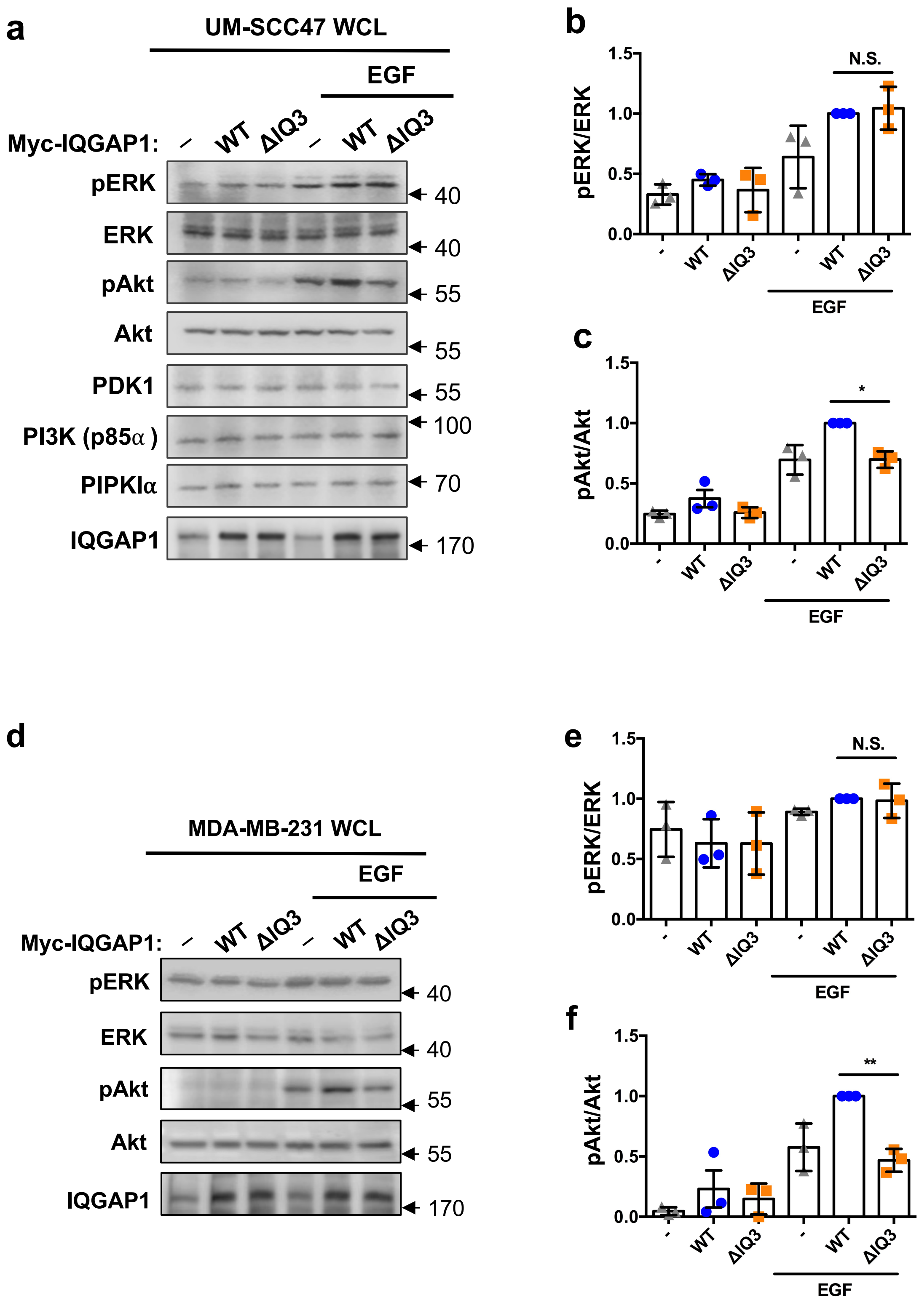

Supplementary Fig. 4 The IQ3 motif in IQGAP1 specifically mediates the PI3K-Akt pathway in UM-SCC47 and MDA-MB-231 cells.

(**a**) EGF stimulated pAkt (S473) and pERK were measured in UM-SCC47 cells. UM-SCC47 cells post 24 h of transient transfection with the Myc-tagged IQGAP1$^{WT/\Delta IQ3}$ constructs were starved for 24 h and then treated with 10 ng/ml EGF for 15 min as indicated. The whole cell lysates were collected and processed for WB. (**b,c**) Quantification of relative pERK and pAkt level in **a**. Unprocessed images of the blots are shown in **Supplementary Fig. 9**. (**d**) EGF stimulated pAkt (S473) and pERK were measured in MDA-MB-231 cells. MDA-MB-231 cells post 24 h of transient transfection with Myc-tagged IQGAP1$^{WT/\Delta IQ3}$ constructs were starved for 24 h and then treated with 10 ng/ml EGF for 15 min as indicated. The whole cell lysates were collected and processed for WB. Unprocessed images of the blots are shown in **Supplementary Fig. 9**. (**e,f**) Quantification of relative pERK and pAkt level in **d**. ***P*<0.01, n=3. Error bars denote SD.

Supplementary Fig. 5

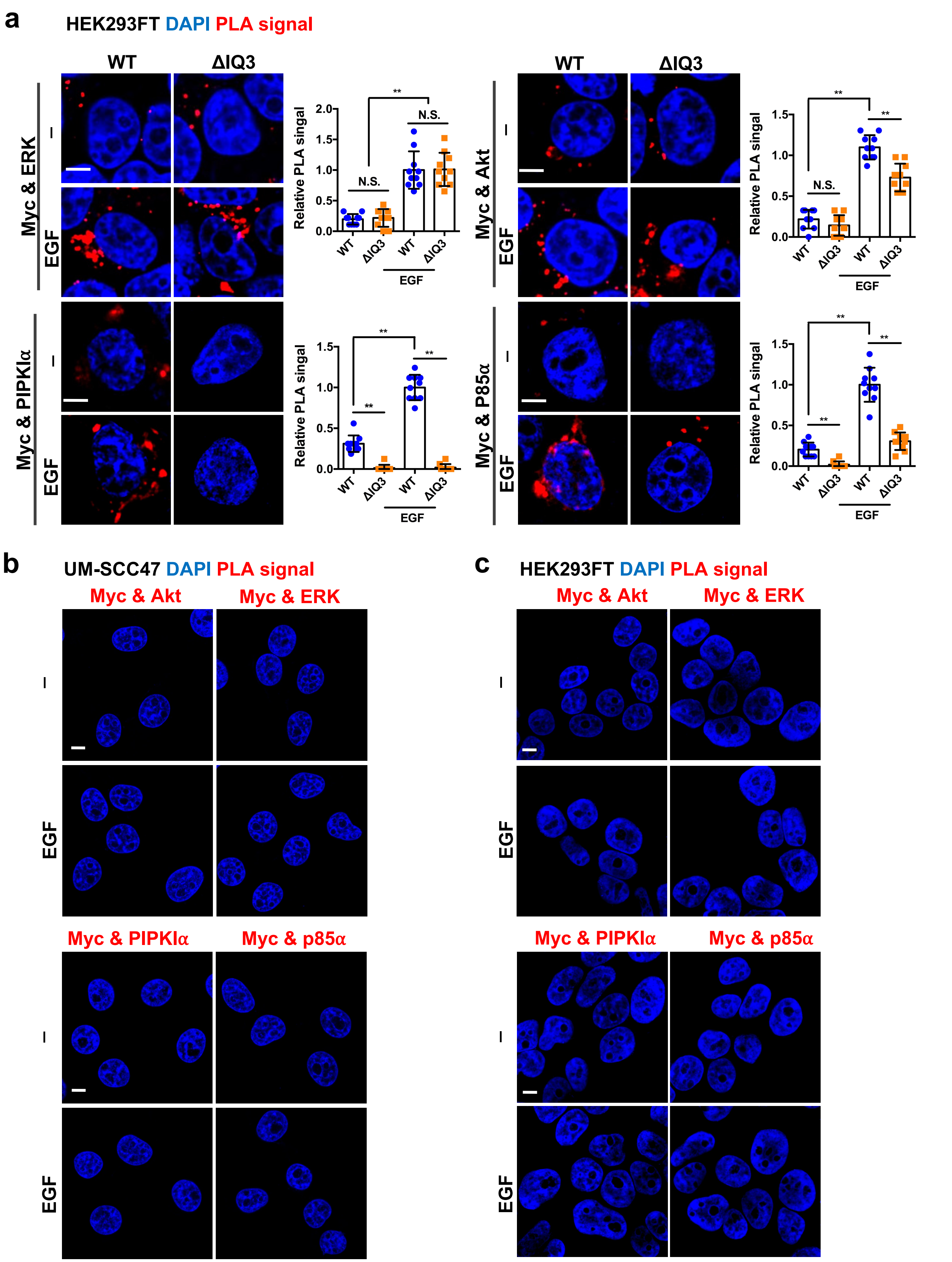

Supplementary Fig. 5 IQ3 deletion in IQGAP1 retains its interaction with ERK but reduces the interaction with PI3K-Akt pathway components through PLA.

(**a**) HEK293FT cells post 24 h of transient transfection with Myc-tagged IQGAP1^{WT} or Myc-tagged IQGAP1$^{\Delta IQ3}$ constructs were starved for 24 h and then treated with 10 ng/ml EGF for 15 min. The cells were fixed and processed for PLA to determine the direct interaction between the Myc-tagged IQGAP1 WT/ΔIQ3 mutant and ERK/Akt/PIPKIα/p85α. **$P<0.01$, $n=10$. Error bars denote SD. Scale bar, 5 µm. (**b,c**) No detectable PLA signal between Myc-tag and ERK/Akt/PIPKIα/p85α in the mock-transfected cells. UM-SCC47 and HEK293FT cells post 24 h of mock transfection were starved for 24 h and then treated with 10 ng/ml EGF for 15 min. The cells were fixed and processed for PLA to determine the direct interaction between Myc-tagged IQGAP1 and ERK/Akt/PIPKIα/p85α.

Supplementary Fig. 6

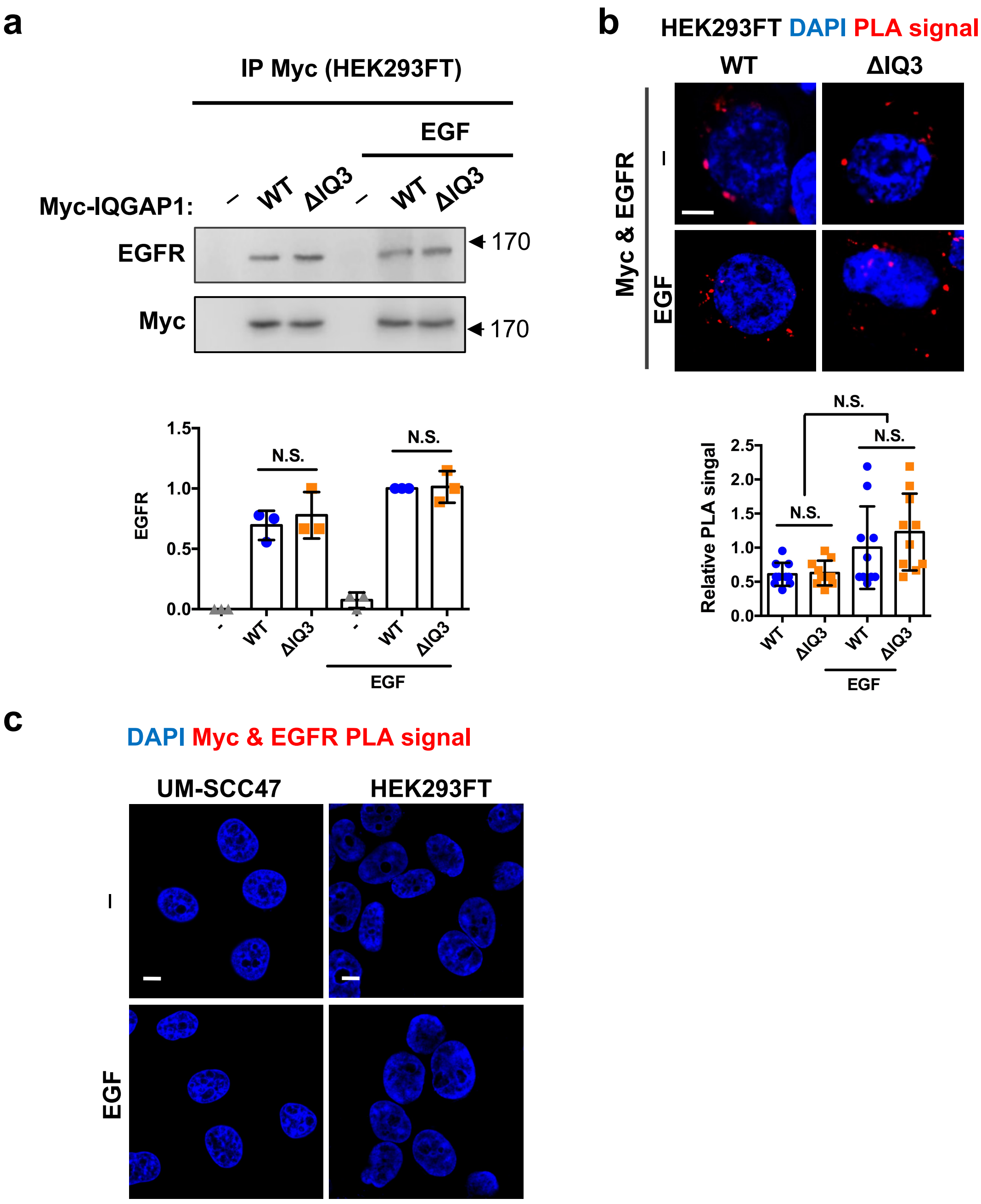

Supplementary Fig. 6 IQ3 deletion in IQGAP1 sustains its interaction with EGFR.
(**a**) IQ3 deletion in IQGAP1 sustained he interaction with EGFR by immunoprecipitation. HEK293FT cells post 24 h of transient transfection with the Myc-tagged IQGAP1$^{WT/\Delta IQ3}$ constructs were starved for 24 h and then treated with 10 ng/ml EGF for 15 min as indicated. The whole cell lysates were collected and processed for immunoprecipitation using anti-Myc antibody conjugated Protein A/G agarose beads. Unprocessed images of the blots are shown in **Supplementary Fig. 9**. **P<0.01, n=3. Error bars denote SD. (**b**) Deletion of IQ3 motif remained the interaction of IQGAP1 with EGFR through PLA. HEK293FT cells post 24 h of transient transfection with Myc-tagged IQGAP1^{WT} or Myc-tagged IQGAP1$^{\Delta IQ3}$ constructs were starved for 24 h and then treated with 10 ng/ml EGF for 15 min. The cells were fixed and processed for PLA to determine the direct interaction between the Myc-tagged IQGAP1 WT/ΔIQ3 mutant and EGFR. **P<0.01, n=10. Error bars denote SD. Scale bar, 5 μm. (**c**) No detectable PLA signal between Myc-tagged IQGAP1 and EGFR in the mock-transfected cells. UM-SCC47 and HEK293FT cells post 24 h of mock transfection were starved for 24 h and then treated with 10 ng/ml EGF for 15 min. The cells were fixed and processed for PLA to determine the direct interaction between Myc-tagged IQGAP1 and EGFR.

Supplementary Fig. 7

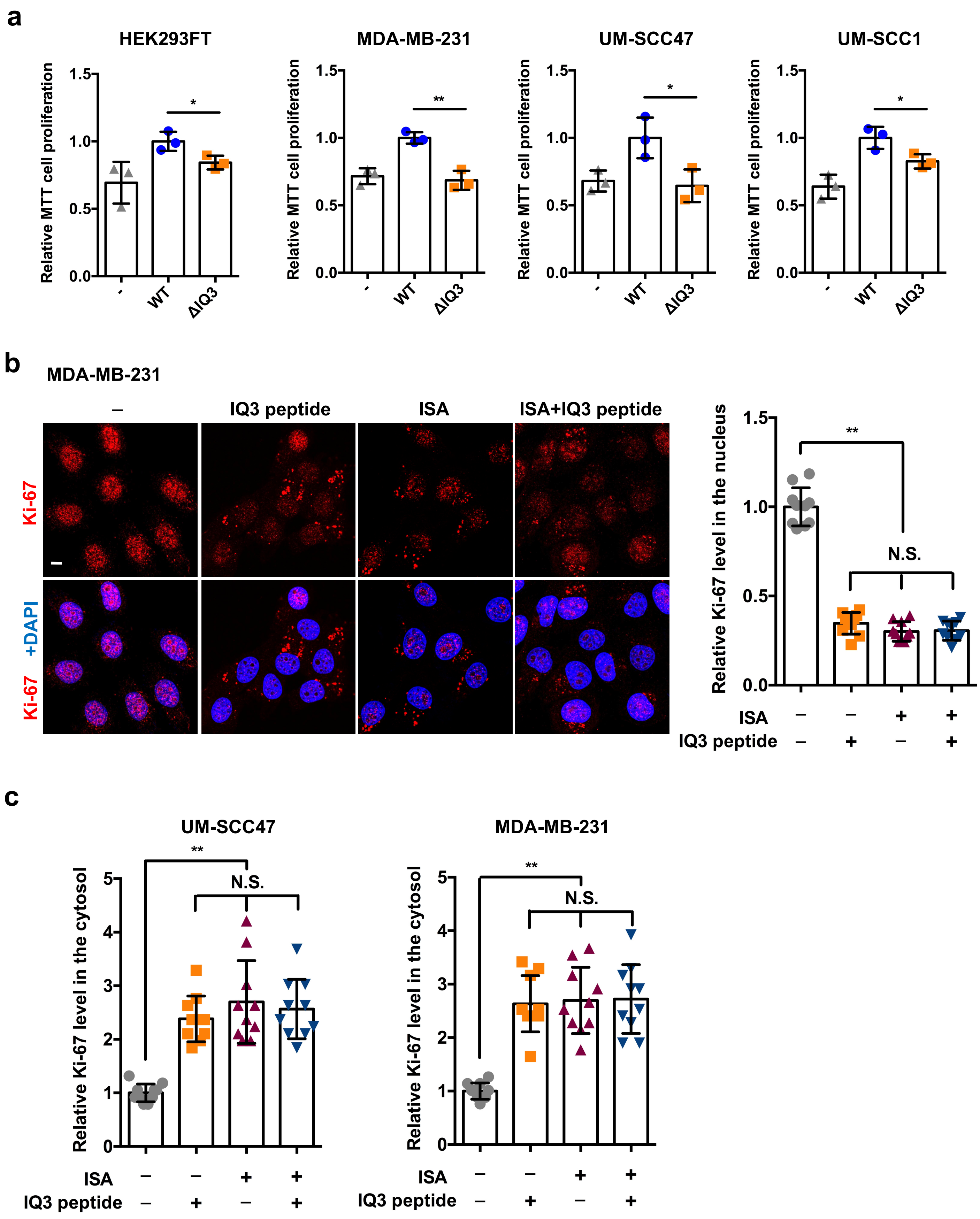

Supplementary Fig. 7 Deleting or blocking IQ3 motif in IQGAP1 reduces its inducible function in cell proliferation.
(**a**) Deletion of IQ3 motif in IQGAP1 reduced its inducible function in cell proliferation through the MTT cell proliferation assay. HEK293FT, MDA-MB-231, UM-SCC47, or UM-SCC1 cells post 48 h of transient transfection with the Myc-tagged $IQGAP1^{WT/\Delta IQ3}$ constructs were processed for MTT cell proliferation assay. $^{**}P<0.01$, n=3. Error bars denote SD. (**b**) IQ3 peptide and PIPKIα inhibitor ISA inhibited cell proliferation in a non-additive manner through the immunofluorescent staining of Ki-67. MDA-MB-231 cells treated with 30 μM IQ3 peptide, 30 μM ISA, or the combination of them for 48 h were processed for immunofluorescent staining against Ki-67. The nuclei were counterstained by DAPI. The images were taken by Leica SP8 confocal microscope. The nuclear Ki-67 level was quantified using ImageJ. $^{**}P<0.01$, n=10. Error bars denote SD. Scale bar, 5 μm. (**c**) IQ3 peptide and PIPKIα inhibitor ISA increased the cytoplasmic level of Ki-67. The cytoplasmic level of Ki-67 in UM-SCC47 cells (Fig. 6d) and MDA-MB-231 cells (Supplementary Fig. 7b) were quantified using ImageJ. $^{**}P<0.01$, n=10. Error bars denote SD.

Supplementary Fig. 8

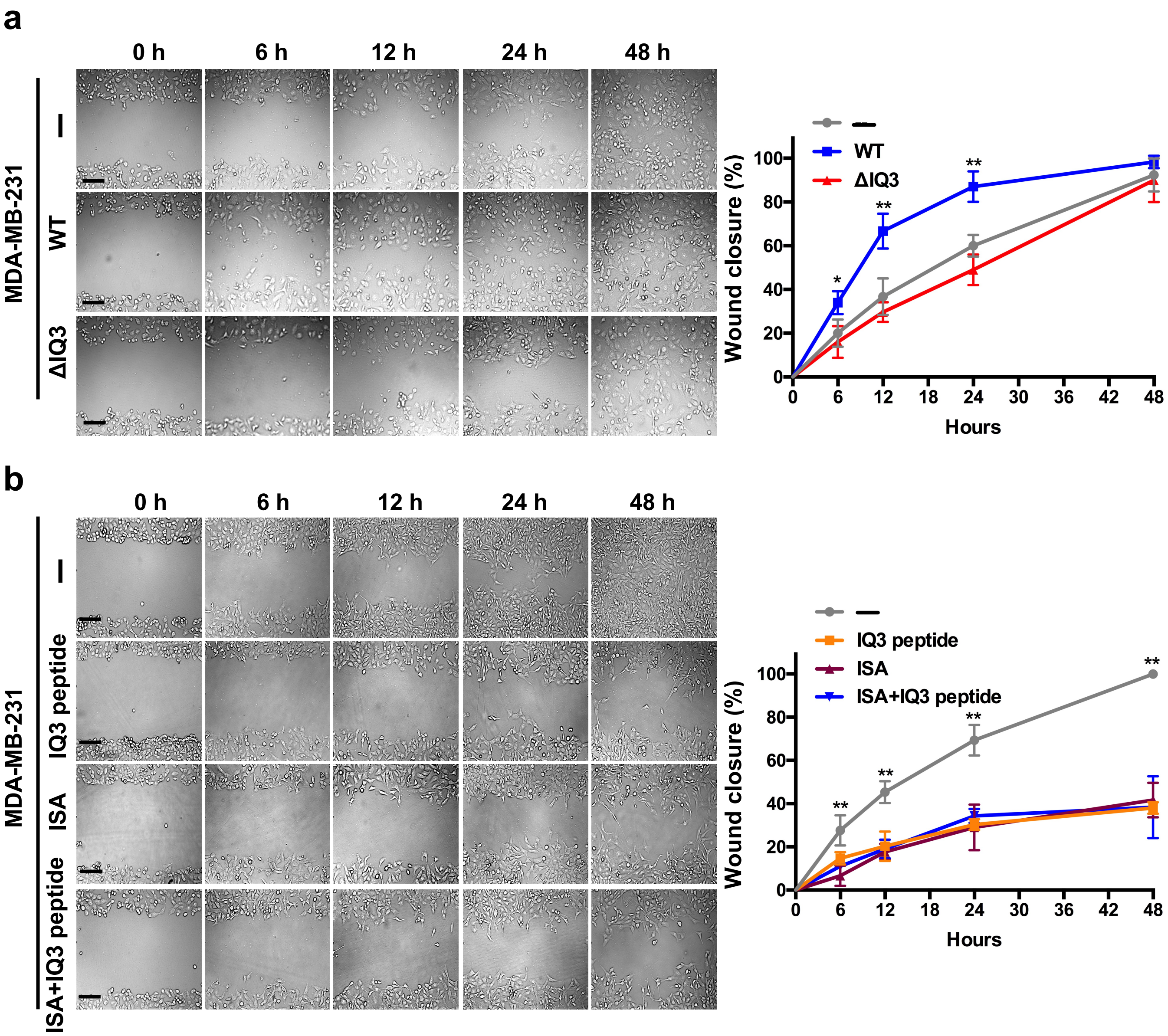

Supplementary Fig. 8 Deleting or blocking IQ3 motif in IQGAP1 reduces its inducible function in MDA-MB-231 cell migration.

(**a**) Deletion of IQ3 motif in IQGAP1 lost its inducible function in cell migration. The MDA-MB-231 cells were seeded and transfected with the Myc-tagged IQGAP1$^{WT/\Delta IQ3}$ for 24 h and then grown to confluence. The cells were starved in serum-free medium for 24 h and then treated with 10 ng/ml EGF. The cellular layer in each plate was scratched using a plastic pipette tip. The migration of the cells at the edge of the scratch was imaged at 0, 6, 12, 24 and 48 h. Scale bar, 100 µm. $^{**}P<0.01$, n=3. Error bars denote SD. (**b**) IQ3 peptide and PIPKIα inhibitor ISA inhibited cell migration in a non-additive manner. The confluent MDA-MB-231 cells were starved in serum-free medium for 24 h and then treated with 30 µM IQ3 peptide, 30 µM ISA, or the combination of them in the presence of 10 ng/ml EGF. The cellular layer in each plate was scratched using a plastic pipette tip. The migration of the cells at the edge of the scratch was imaged at 0, 6, 12, 24 and 48 h. Scale bar, 100 µm. $^{**}P<0.01$, n=3. Error bars denote SD.

Supplementary Fig. 9 Unprocessed gel images

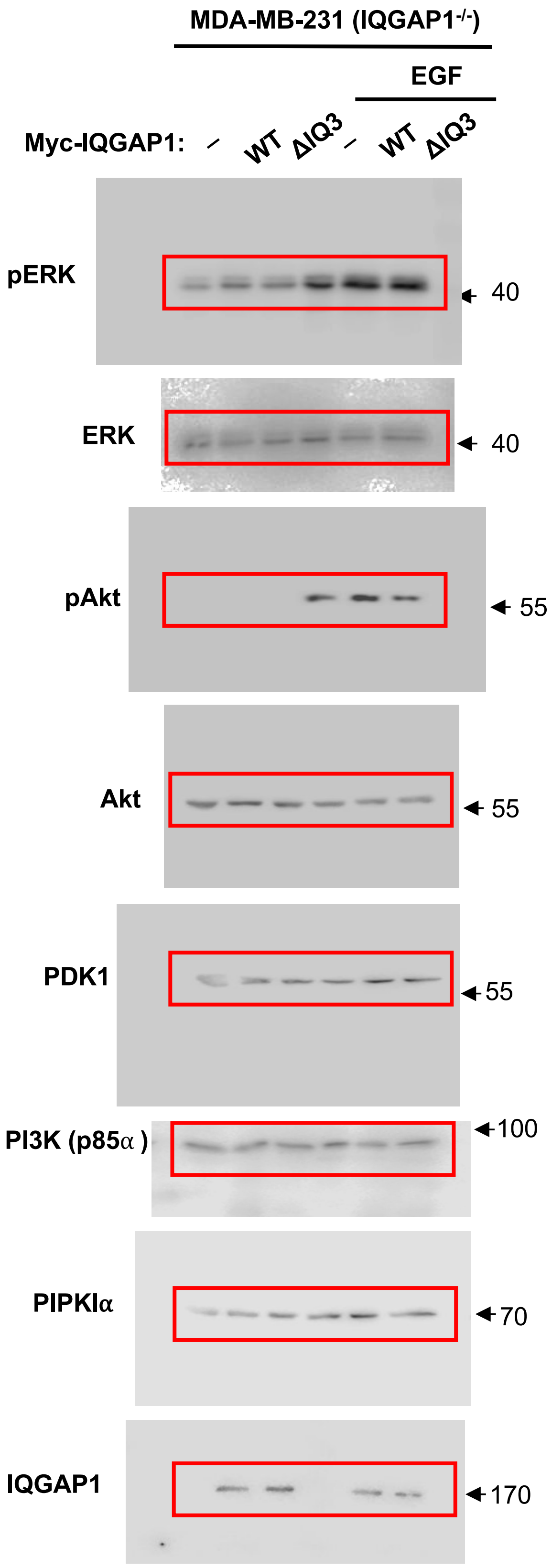

Fig. 1f

Supplementary Fig. 9 (Continued)

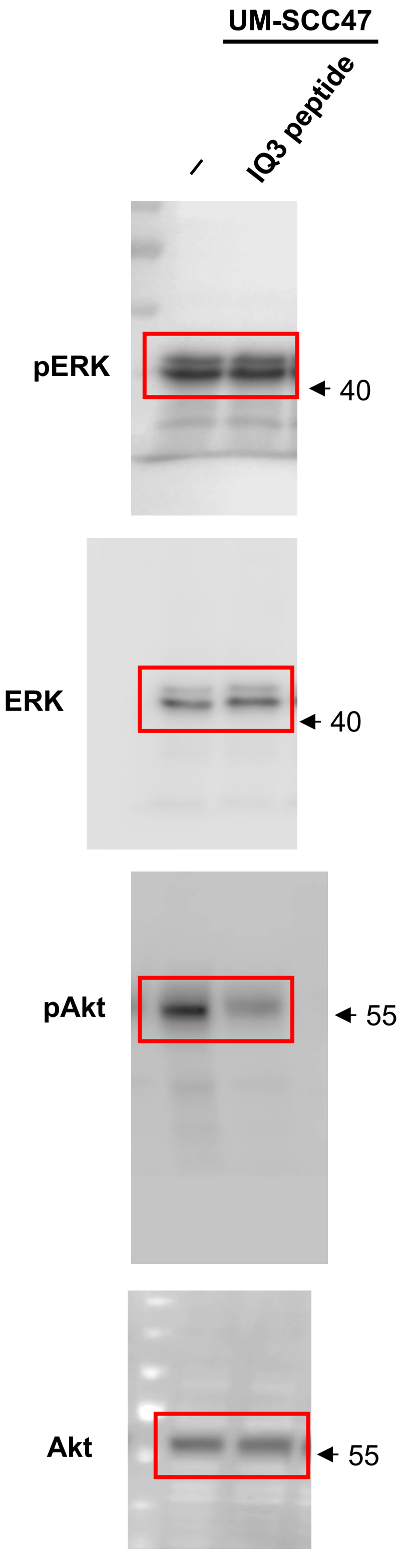

Fig. 2a

Supplementary Fig. 9 (Continued)

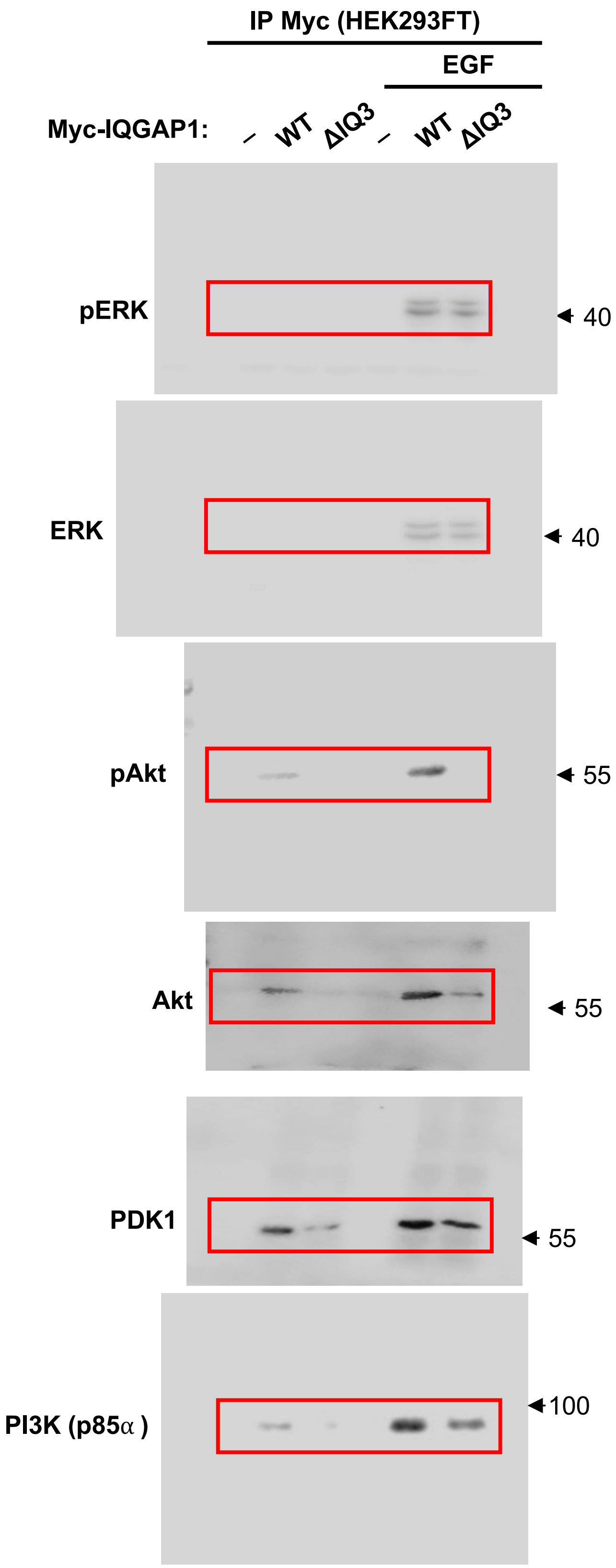

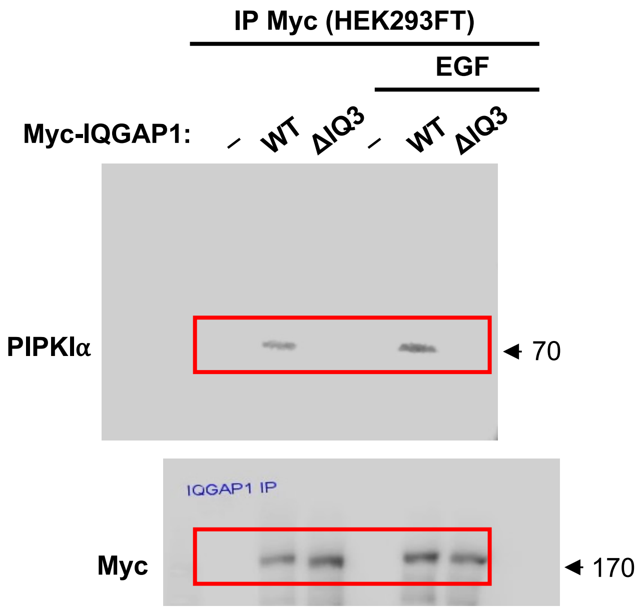

Fig. 3a

Supplementary Fig. 9 (Continued)

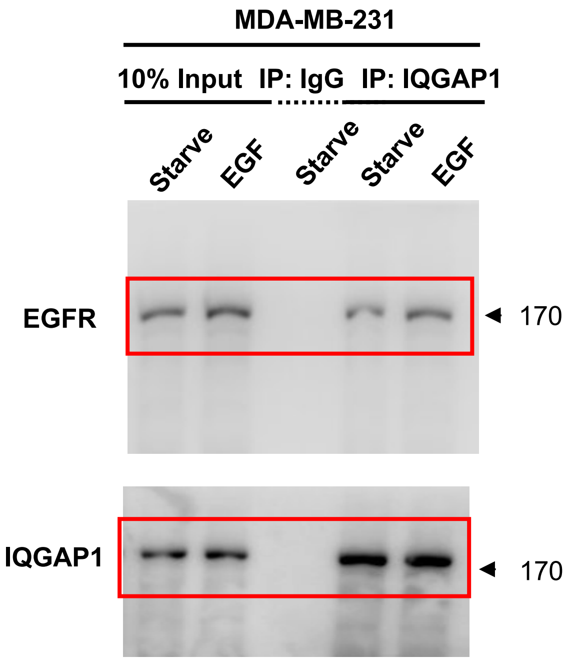

Fig. 4c

Supplementary Fig. 9 (Continued)

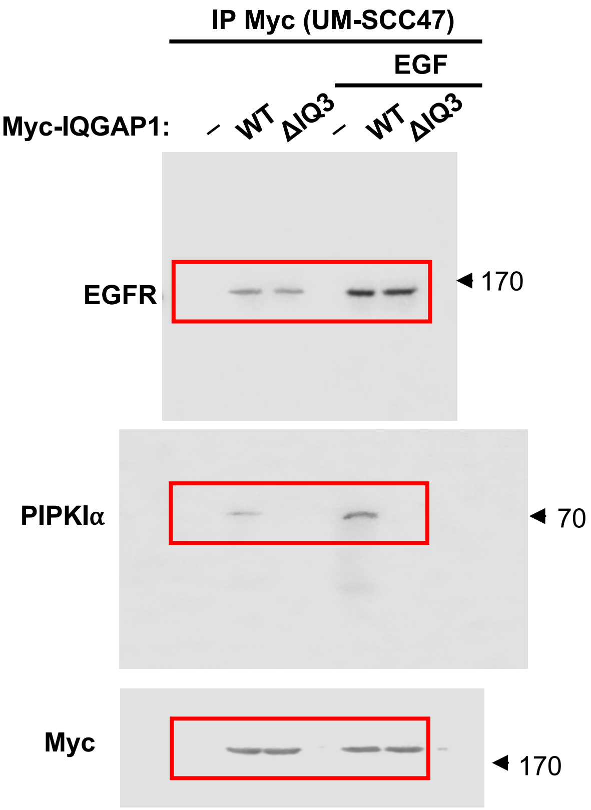

Fig. 4d

Supplementary Fig. 9 (Continued)

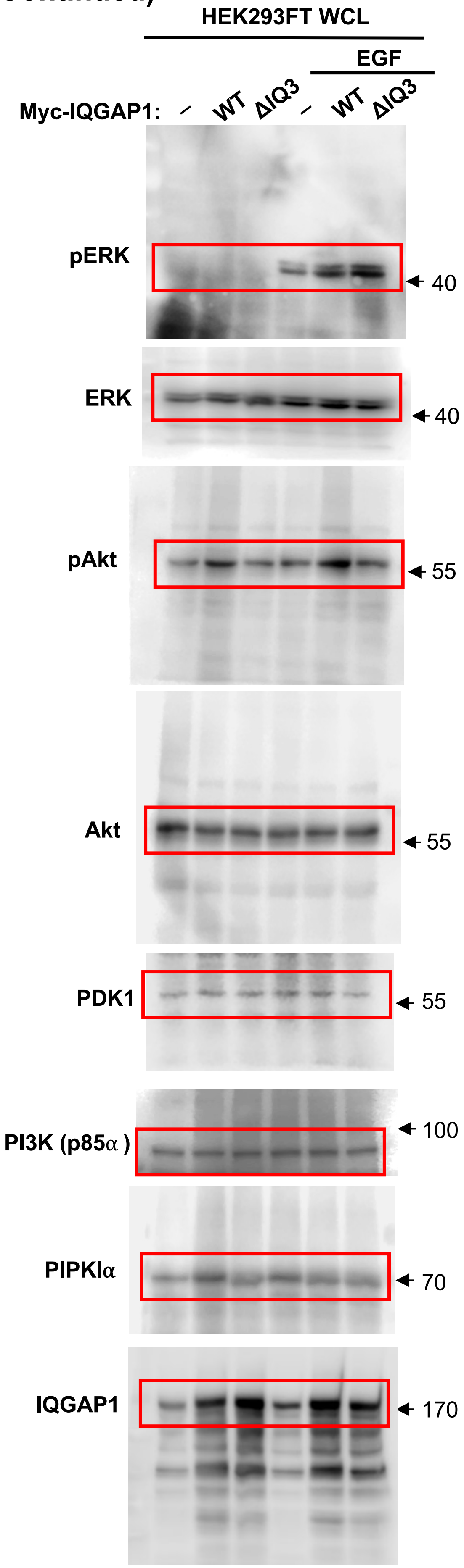

Supplementary Fig. 3a

Supplementary Fig. 9 (Continued)

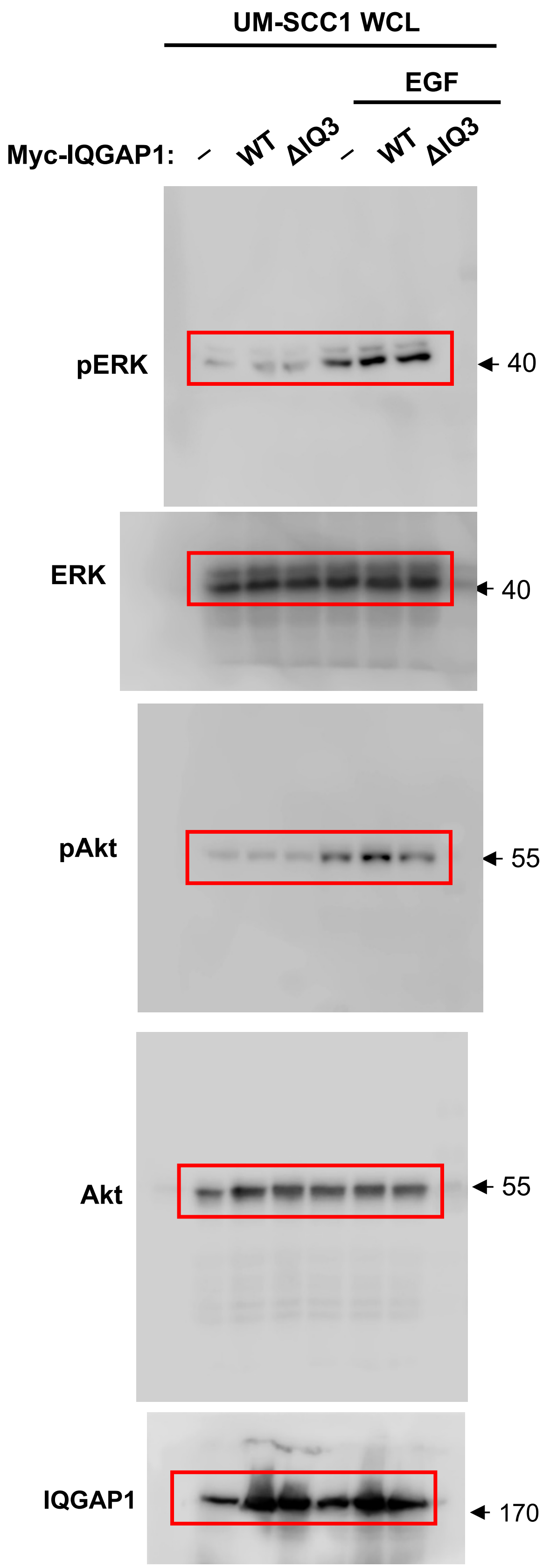

Supplementary Fig. 3d

Supplementary Fig. 9 (Continued)

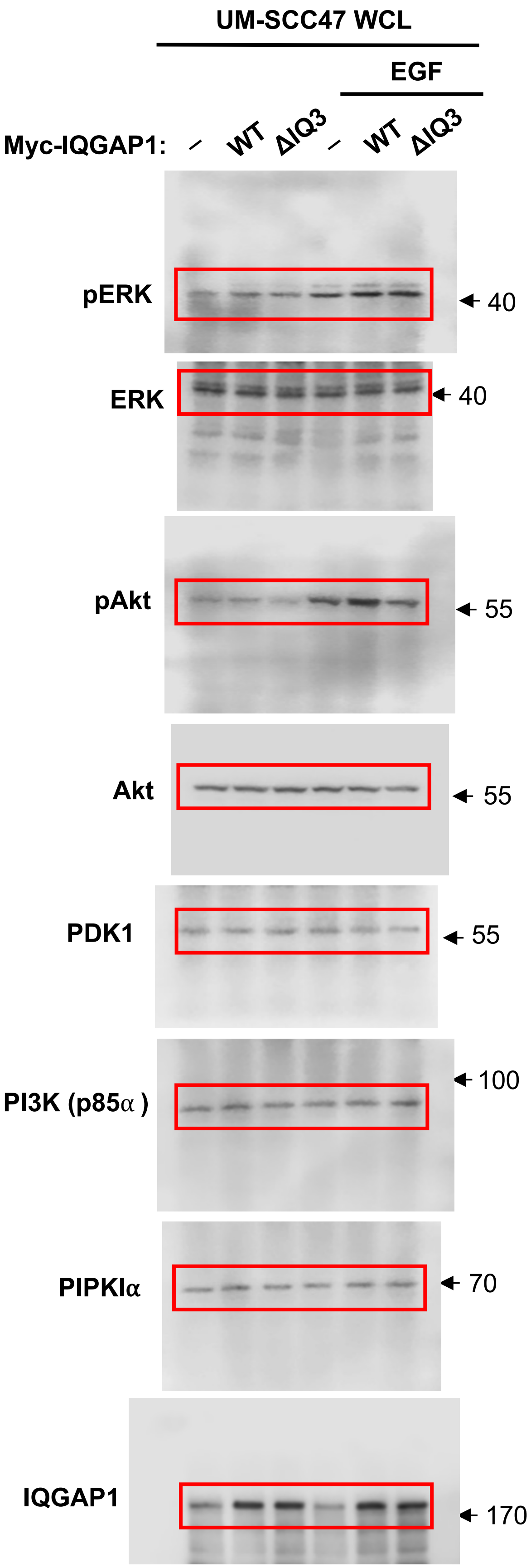

Supplementary Fig. 4a

Supplementary Fig. 9 (Continued)

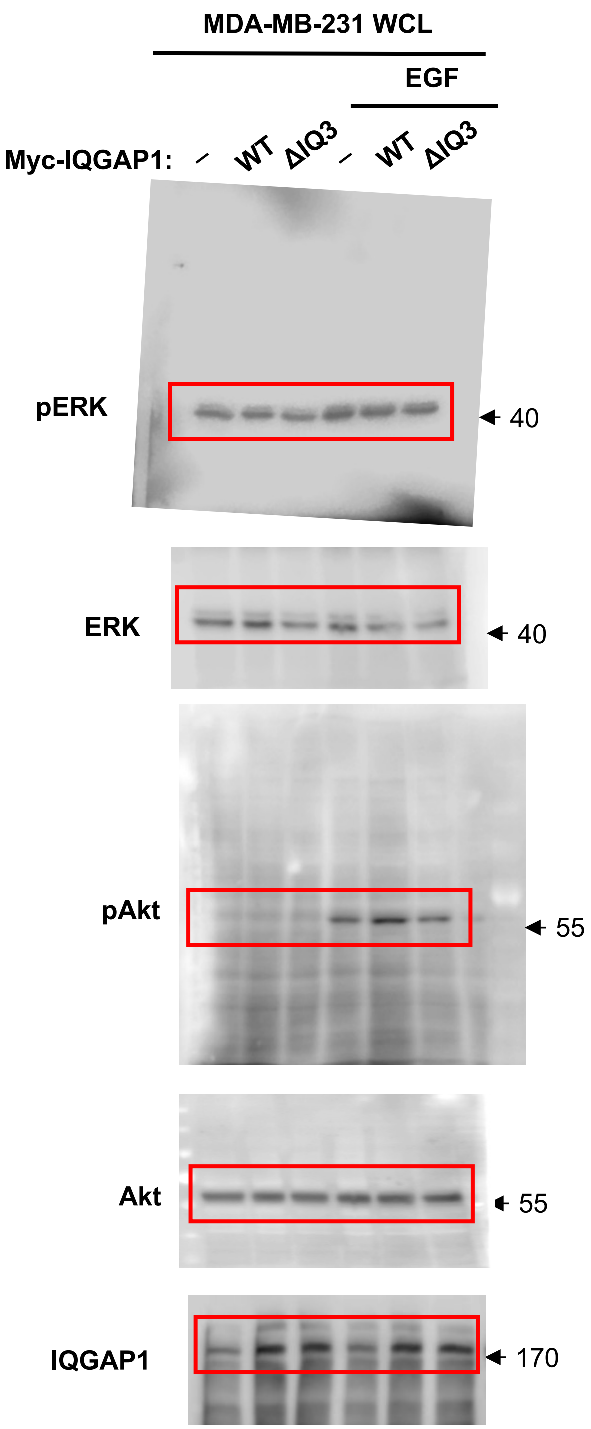

Supplementary Fig. 4d

Supplementary Fig. 9 (Continued)

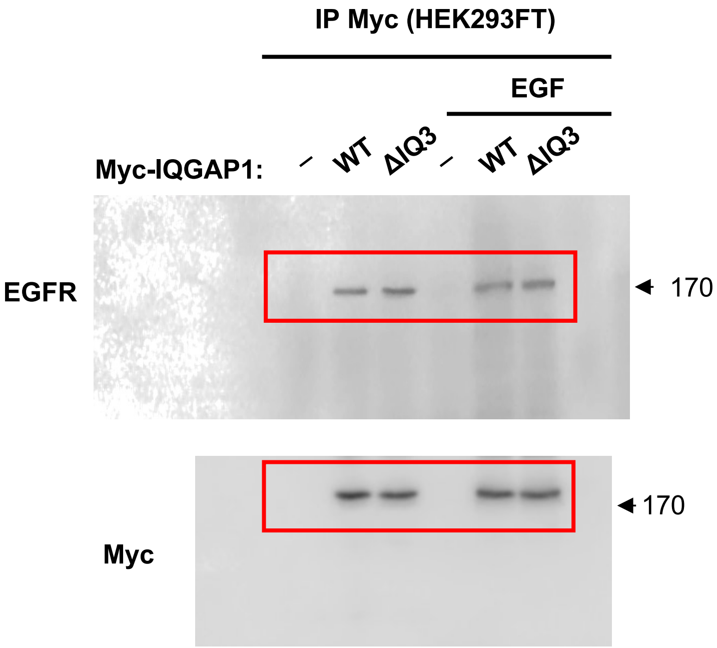

Supplementary Fig. 6a
